# The deep phylogeny of jumping spiders (Araneae, Salticidae)

**DOI:** 10.3897/zookeys.440.7891

**Published:** 2014-09-15

**Authors:** Wayne P. Maddison, Daiqin Li, Melissa Bodner, Junxia Zhang, Xin Xu, Qingqing Liu, Fengxiang Liu

**Affiliations:** 1 Beaty Biodiversity Museum and Department of Botany, University of British Columbia, Vancouver, British Columbia, V6T 1Z4 Canada; 2 Department of Zoology, University of British Columbia, Vancouver, British Columbia, V6T 1Z4 Canada; 3 Centre for Behavioural Ecology & Evolution, College of Life Sciences, Hubei University, Wuhan 430062, Hubei, China; 4 Department of Biological Sciences, National University of Singapore, 14 Science Drive 4, Singapore 117543

**Keywords:** Jumping spiders, Salticidae, phylogeny, systematics

## Abstract

In order to resolve better the deep relationships among salticid spiders, we compiled and analyzed a molecular dataset of 169 salticid taxa (and 7 outgroups) and 8 gene regions. This dataset adds many new taxa to previous analyses, especially among the non-salticoid salticids, as well as two new genes – *wingless* and myosin heavy chain. Both of these genes, and especially the better sampled *wingless*, confirm many of the relationships indicated by other genes. The cocalodines are placed as sister to lapsiines, in a broader clade with the spartaeines. Cocalodines, lapsiines, and spartaeines are each supported as monophyletic, though the first two have no known morphological synapomorphies. The lyssomanines appear to be non-monophyletic, of three separate groups: (1) *Lyssomanes* plus *Chinoscopus*, (2) *Onomastus*, and (3) the remainder of Old World species. Several previously-inferred relationships continue to be supported: hisponines as sister to the Salticoida, Amycoida as sister to the remaining Salticoida, and Saltafresia as monophyletic. The relationship of *Salticus* with *Philaeus* and relatives is now considered well enough corroborated to move the latter into the subfamily Salticinae. A new clade consisting of the Plexippoida + Aelurilloida + Leptorchesteae + Salticinae is recognized. *Nungia* is found to be an astioid, and *Echeclus*, *Gedea* and *Diplocanthopoda* to be hasariines. The euophryines are corroborated as monophyletic. The agoriines *Agorius* and *Synagelides* are salticoids, within the sister group to amycoids, but their further placement is problematical, perhaps because of their nuclear ribosomal genes’ high GC bias, as also seen in the similarly problematic *Eupoa*.

## Introduction

Salticid spiders, remarkable for their excellent vision (Land 1969, Blest et al. 1990), include more than 5000 species (Platnick 2014) with a great diversity of body forms and behaviours. While this diversity has long resisted phylogenetic organization, recent molecular studies (Maddison and Hedin 2003, Su et al. 2007, Maddison et al. 2008, Bodner and Maddison 2012, Zhang and Maddison 2013), aided by compilations of morphological taxonomic knowledge (Prószyński 2013) have resolved much of the phylogenetic structure of the family. One of the best-supported clades is the Salticoida, recognized by both morphological and molecular data (Maddison 1996, Maddison and Hedin 2003) and containing about 95% of the known species in the family. Within the Salticoida, large groups such as the Amycoida, Astioida, Marpissoida and Plexippoida are well-corroborated (Maddison and Hedin 2003, Maddison et al. 2008). However, many of the deeper relationships of salticoids have been poorly resolved. Outside the Salticoida are the spartaeines, lyssomanines, and hisponines, showing ancestral features like limited tracheal systems, complex palpi, and the retention of a tarsal claw on the female palp. These non-salticoids (often called “basal salticids”) have been studied phylogenetically (Su et al. 2007), but with limited taxon sampling.

In this work we attempt to resolve more firmly the basic structure of the family by increasing the taxon sampling, especially among non-salticoid salticids, and by using additional genes. Two of the genes, *wingless* and myosin heavy chain, are new to salticid molecular phylogenetics. By building a dataset that has a greater number of genes among selected species, we hoped to obtain a phylogenetic resolution with stronger confidence.

## Methods

### Taxon sampling

Taxa included in the analysis are 169 species of salticids and representatives of four dionychan families as outgroups (Table 1, Suppl. material 1). Based on previous phylogenetic work (Maddison and Hedin 2003, Maddison et al. 2008, Bodner and Maddison 2012, Zhang and Maddison 2013, in press), about 70 species of salticids from the major clade Salticoida were selected because they would represent most known major lineages, and because several genes are available for each (Table 1, Suppl. material 1). In addition, a few salticoids were added because their placement was unclear: *Agorius*, *Diplocanthopoda*, *Echeclus*, *Gedea*, *Nungia*, *Phaulostylus*, and *Synagelides*.

**Table 1.** Specimens and sequences used in phylogenetic analyses, with GenBank numbers indicated. * marks previously published sequences. Specimen localities given in Suppl. material 1.

	Reference	28s	18s	*wingless*	myosin HC	actin 5c	histone 3	CO1	16sND1
**Outgroups**									
Anyphaenidae: *Hibana* sp.	s318	AY297295*	KM033091		KM032961	KM032929		AY297422*	AY297295 / AY297358*
Gnaphosidae: *Cesonia* sp.	s319	AY297293; EF201663*		KM032996		EU522700*	DQ665720*	AY297420*	AY296711 / AY297356*
Miturgidae: *Cheiracanthium* sp.	s321	AY297294; EF201664*		KM032997		KM032928		AY297421*	AY296712 / AY297357*
Oxyopidae: *Oxyopes birmanicus* Thorell, 1887	Su et al. 2007	EF419032 / EF419065*	EF418998*				EF419126*	EF419097*	EF418969 / EF419150*
Philodromidae: *Philodromus alascensis* Keyserling, 1884	GR011	KM033130	KM033092	KM032998	KM032962				
Thomisidae: *Misumenops nepenthicola* (Pocock, 1898)	Su et al. 2007	EF419029 / EF419062*	EF418996*				EF419123*	EF419094*	EF418967 / EF419148*
Thomisidae: *Xysticus* sp.	s316	AY297296; EF201665*	KM033093			EU522701*	DQ665704*	AY297296*	AY296714 / AY297359*
**Lyssomanines**									
*Asemonea sichuanensis* Song & Chai, 1992	SC-03-0055		EF418986*					EF419082*	
*Asemonea sichuanensis* Song & Chai, 1992	MRB084	KM033131				KM032931			
*Asemonea* cf. *stella* Wanless, 1980	MRB083	JX145767*	KM033094			KM032930		JX145686*	
*Asemonea tenuipes* (O. P.-Cambridge, 1869)	d186	KM033132	KM033095	KM032999	KM032963	KM032932			
*Chinoscopus* cf. *flavus* (Peckham, Peckham & Wheeler, 1889)	d273	KM033133	KM033096						KM032888
*Goleba lyra* Maddison & Zhang, 2006	d051	DQ665768*	KM033097	KM033000		EU522709*	DQ665707*	DQ665755*	
*Lyssomanes amazonicus* Peckham & Wheeler, 1889	ECU11-6112	KM033134							KM032889
*Lyssomanes antillanus* Peckham & Wheeler, 1889	d298	KM033135		KM033001					
*Lyssomanes* cf. *benderi* Logunov, 2002	ECU11-5402	KM033136							KM032890
*Lyssomanes* cf. *jemineus* Peckham & Wheeler, 1889	ECU11-5682	KM033137							KM032891
*Lyssomanes longipes* (Taczanowski, 1871)	MRB086	KM033138				KM032933		KM033208	KM032892
*Lyssomanes pauper* Mello-Leitão, 1945	d297	KM033139		KM033002					
*Lyssomanes taczanowskii* Galiano, 1980	ECU11-4193	KM033141							KM032894
*Lyssomanes tenuis* Peckham & Wheeler, 1889	ECU11-4869	KM033142							KM032895
*Lyssomanes viridis* (Walckenaer, 1837)	s160	AY297231*						AY297360*	AY296652 / AY297297*
*Lyssomanes viridis* (Walckenaer, 1837)	d129		KM033098	KM033003		EU522715*	DQ665715*		
*Lyssomanes* sp. [Esmeraldas]	d408	KM033140							KM032893
*Onomastus nigrimaculatus* Zhang & Li, 2005	Su et al. 2007	EF419031 / EF419064*	EF418997*				EF419125*	EF419096*	EF418968 / EF419149*
*Onomastus* sp. [Guangxi]	MRB085	JX145768*	KM033099	KM033004	KM032964	KM032934		JX145687*	JX145910*
*Pandisus* cf. *decorus* Wanless, 1980	d303	KM033143		KM033005					
**Cocalodines**									
*Allococalodes madidus* Maddison, 2009	d236	KM033144		KM033006					KM032896
*Cocalodes longicornis* Wanless, 1982	d291	KM033145		KM033007		KM032935			KM032897
*Cocalodes macellus* (Thorell, 1878)	d230	KM033146	KM033100	KM033008		KM032936		KM033209	
*Cucudeta gahavisuka* Maddison, 2009	d234	KM033147		KM033009					KM032898
*Cucudeta zabkai* Maddison, 2009	d235	KM033148		KM033010	KM032965				KM032899
*Tabuina* aff. *baiteta* Maddison, 2009	d313	KM033149		KM033011					
*Tabuina rufa* Maddison, 2009	d232	KM033151		KM033013					KM032900
*Tabuina* aff. *rufa* Maddison, 2009	d312	KM033150		KM033012					
*Tabuina varirata* Maddison, 2009	d233	KM033152		KM033014					KM032901
*Yamangalea frewana* Maddison, 2009	d231	KM033153		KM033015					KM032902
**Spartaeines**									
*Brettus* cf. *adonis* Simon, 1900	SWK12-4323	KM033154							
*Brettus* sp. [Yunnan]	LiD-026-053-05	KM033155^S^	KM033101^S^				KM033195^S^		
cf. *Phaeacius* sp. [Sarawak]	SWK12-3728	KM033156							
*Cocalus murinus* Simon, 1899	LiD-013-027-05	EF419019 / EF419053*	EF418988*				EF419116*	EF419084*	EF418959 / EF419140*
*Cyrba algerina* (Lucas, 1846)	Su et al. 2007	EF419021 / EF419054*	EF418989*					EF419086*	EF418961 / EF419142*
*Cyrba lineata* Wanless, 1984	MRB106	JX145792*		KM033016	KM032966	KM032937		JX145704*	
*Cyrba ocellata* (Kroneberg, 1875)	Su et al. 2007		EF418990*					EF419087*	EF418962 / EF419143*
*Cyrba ocellata* (Kroneberg, 1875)	MRB104	KM033157							
*Cyrba* sp. [Kenya]	Su et al. 2007	EF419023 / EF419056*	EF418991*					EF419088*	
*Gelotia* cf. *bimaculata* Thorell, 1890	d250	KM033158		KM033017		KM032938			
*Gelotia syringopalpis* Wanless, 1984	Su et al. 2007	EF419024 / EF419057*					EF419118*		
*Gelotia syringopalpis* Wanless, 1984	MRB105			KM033019				KM033212	KM032903
*Gelotia* sp. [Guangxi]	MRB199			KM033018		KM032939		KM033210	
*Gelotia* sp. [Yunnan]	LiD002-053-05		KM033102^S^				KM033196^S^	KM033211^S^	
*Holcolaetis vellerea* Simon, 1910	Su et al. 2007	EF419025 / EF419058*	EF418992*				EF419119*	EF419090*	EF418963 / EF419144*
*Holcolaetis* cf. *zuluensis* Lawrence, 1937	d036	DQ665770*	KM033103			EU522711*	DQ665721*	DQ665757*	
*Meleon* aff. *kenti* (Lessert, 1925)	d287	KM033159				KM032940			
*Mintonia mackiei* Wanless, 1984	SWK12-4202	KM033161							
*Mintonia* cf. *melinauensis* Wanless, 1984	d441	KM033160							
*Mintonia ramipalpis* (Thorell, 1890)	SWK12-1442	KM033162							
*Mintonia silvicola* Wanless, 1987	d104			KM033020					KM032904
*Mintonia silvicola* Wanless, 1987	SWK12-1653	KM033163							
*Mintonia silvicola* Wanless, 1987	Su et al. 2007		EF418995*				EF419122*	EF419093*	
*Mintonia tauricornis* Wanless, 1984	d249	KM033164		KM033021		KM032941			KM032905
*Neobrettus tibialis* (Prószyński, 1978)	LiD-001-055-05	EF419030 / EF419063*					EF419124*	EF419095*	
*Neobrettus* sp. [Sarawak]	SWK12-1040	KM033165							
*Paracyrba wanlessi* Zabka & Kovac, 1996	Su et al. 2007	EF419033 / EF419066*	EF418999*					EF419098*	
*Phaeacius lancearius* (Thorell, 1895)	d111	DQ665775*		KM033022				DQ665759*	
*Phaeacius malayensis* Wanless, 1981	Su et al. 2007	EF419034 / EF419067*	EF419000*					EF419099*	EF418970 / EF419151*
*Phaeacius* sp. [Guangxi]	LQ-24-06	KM033166^S^	KM033104^S^					KM033213^S^	KM032906^S^
*Phaeacius* sp. [Hainan]	Su et al. 2007	EF419035 / EF419068*	EF419001*						EF418971 / EF419152*
*Phaeacius* sp. [Sarawak]	SWK12-4541	KM033167							
*Portia africana* (Simon, 1886)	Su et al. 2007	EF419037 / EF419069*	EF419003*				EF419128*	EF419101*	
*Portia crassipalpis* (Peckham & Peckham, 1907)	SWK12-2354	KM033168							
*Portia fimbriata* (Doleschall, 1859)	LiD-001-04	EF419038 / EF419070*	EF419004*				EF419129*	EF419102*	EF418973 / EF419154*
*Portia heteroidea* Xie & Yin, 1991	Su et al. 2007	EF419039 / EF419071*	EF419005*				EF419130*	EF419103*	EF418974 / EF419155*
*Portia jianfeng* Song & Zhu, 1998	Su et al. 2007	EF419040 / EF419072*	EF419006*					EF419104*	EF418975 / EF419156*
*Portia labiata* (Thorell, 1887)	S206	AY297232*						AY297361*	AY296653 / AY297298*
*Portia* cf. *schultzi* Karsch, 1878	d131	DQ665776*	KM033105	KM033023	KM032967	EU522718*	DQ665708*		
*Portia quei* Zabka, 1985	Su et al. 2007	EF419042 / EF419074*	EF419008*				EF419132*	EF419106*	EF418977 / EF419158*
*Portia taiwanica* Zhang & Li, 2005	MRB103	KM033169				KM032942		KM033214	KM032907
*Portia* sp. [Sichuan]	SC-03-0011	EF419043 / EF419075*	EF419009*				EF419133*		EF418978 / EF419159*
*Sonoita lightfooti* Peckham & Peckham, 1903	d226	KM033170						KM033215	
*Sonoita* aff. *lightfooti* Peckham & Peckham, 1903	MRB200	JX145791*						JX145705*	JX145927*
*Sparbambus gombakensis* Zhang, Woon & Li, 2006	d251	KM033171		KM033024		KM032943			
*Spartaeus jianfengensis* Song & Chai, 1991	Su et al. 2007	EF419045 / EF419076*	EF419011*					EF419109*	EF418980 / EF419161*
*Spartaeus platnicki* Song, Chen & Gong, 1991	SC-03-069	EF419046 / EF419077*	EF419012*				EF419135*	EF419110*	EF418981 / EF419162*
*Spartaeus spinimanus* (Thorell, 1878)	S199							KM033216	KM032908
*Spartaeus thailandicus* Wanless, 1984	BV-004	EF419047 / EF419078*	EF419013*				EF419136*	EF419111*	EF418982 / EF419163*
*Spartaeus uplandicus* Barrion & Litsinger, 1995	S185/S186	AY297233*						AY297363*	AY296655*
*Spartaeus wildtrackii* Wanless, 1987	Su et al. 2007	EF419048 / EF419079*	EF419014*				EF419137*	EF419112*	EF418983 / EF419164*
*Taraxella* sp. [Johor]	d246	KM033172				KM032944			KM032909
*Taraxella* sp. [Pahang]	d248	KM033173				KM032945	KM033197		
*Taraxella* sp. [Pahang]	LiD-001-003-06		KM033106^S^					KM033217^S^	KM032910^S^
*Yaginumanis wanlessi* Zhang & Li, 2005	Su et al. 2007	EF419050 / EF419081*	EF419016*				EF419139*	EF419114*	EF418985 / EF419166*
**Lapsiines**									
*Galianora bryicola* Maddison, 2006	d124	DQ665771*	DQ665741*	KM033025		EU522706*	DQ665717*	DQ665758*	DQ665727*
*Galianora sacha* Maddison, 2006	d116	DQ665766*	DQ665734*	KM033026	KM032968	EU522707*	DQ665716*	DQ665754*	
*Lapsias canandea* Maddison, 2012	d442	KM033174							
*Lapsias guamani* Maddison, 2012	UBC-SEM AR00191	KM033175		KM033027					
*Lapsias lorax* Maddison, 2012	UBC-SEM AR00194	KM033176		KM033028					
*Soesiladeepakius lyra* Ruiz & Maddison, 2012	GR130	JQ312077		KM033029		JQ312074*			JQ312079*
*Thrandina bellavista* Maddison, 2012	d396	KM033177		KM033030					
*Thrandina cosanga* Maddison, 2012	d395	KM033178							
*Thrandina parocula* Maddison, 2006	d123	DQ665779*	KM033107			EU522720*	DQ665718*	DQ665761*	DQ665726*
*Thrandina parocula* Maddison, 2006	d394			KM033031	KM032969				
**Eupoa**									
*Eupoa nezha* Maddison & Zhang, 2007	d220/MRB102	EF201648*	EF201666*	KM033032				EF201668*	EF201667*
**Hisponines**									
cf. *Tomocyrba* sp. [Madagascar]	d305	KM032881*							
*Hispo macfarlanei* Wanless, 1981	d404	KM032882*			KM032970				
*Hispo* sp. [Madagascar]	d309	KM032883*							
*Jerzego* cf. *alboguttatus* Simon, 1903	SWK12-4787	KM032884*							
*Jerzego corticicola* Maddison, 2014	SWK12-2900	KM032885*							KM032887*
*Massagris contortuplicata* Wesolowska & Haddad, 2013	d082	DQ665772*	KM033108	KM033033			DQ665705*		DQ665722*
*Massagris schisma* Maddison & Zhang, 2006	d081	DQ665762*	KM033109	KM033034					DQ665728*
*Tomobella andasibe* (Maddison & Zhang, 2006)	d127	DQ665780*	DQ665752*	KM033035			KM033198		DQ665725*
*Tomocyrba* sp. [Madagascar]	d306	KM032886*							
*Tomomingi* sp. [Gabon]	MRB243	JX145764*	KM033110	KM033036	KM032971	JX145850*		JX145684*	
**Salticoida**									
**Agoriines**									
*Agorius constrictus* Simon, 1901	d172					KM032953			
*Agorius constrictus* Simon, 1901	d213		KM033119	KM033072					KM032921
*Agorius* sp. [Selangor]	d299	KM033189		KM033073					
*Synagelides* cf. *lushanensis* Xie & Yin, 1990	d214			KM033074					
*Synagelides* cf. *palpalis* Zabka, 1985	MRB050								KM032922
*Synagelides* cf. *palpalis* Zabka, 1985	d225	KM033190						KM033226	
**Amycoids**									
*Cotinusa* sp. [Ecuador]	MRB024	JX145746*	KM033120	KM033075	KM032987	JX145832*		JX145671*	JX145896*
*Hurius vulpinus* Simon, 1901	S213	AY297239*						AY297368*	AY296662 / AY297306*
*Hurius* cf. *vulpinus* Simon, 1901	d156			KM033076		EU522712*	KM033203		
*Hypaeus* aff. *miles* Simon, 1900 [Ecuador]	d130	EU815499*	KM033121	KM033077	KM032988	EU522702*			KM032923
*Sarinda cutleri* (Richman, 1965)	MRB193	JX145744*		KM033078		KM032954		JX145669*	JX145895*
*Sitticus floricola palustris* (Peckham & Peckham, 1883)	d030	DQ665778*	KM033122	KM033079	KM032989		KM033204	DQ665760*	DQ665729*
**Astioids**									
*Arasia mollicoma* (L. Koch, 1880)	d046	EU815483*	EU815532*		KM032990	JX145834*	KM033205	EU815598*	EU815550*
*Helpis minitabunda* (L. Koch, 1880)	d265		KM033123	KM033080	KM032991	KM032955		KM033227	
*Ligurra latidens* (Doleschall, 1859)	d175	JX145749*		KM033081		JX145835*			JX145898*
*Ligurra latidens* (Doleschall, 1859)	LiD-001-027-05		EF418993*				EF419120*	EF419091*	
*Mopsus mormon* Karsch, 1878	d018	EU815470*	EU815529*	KM033082		JX145836*	KM033206	EU815586*	
*Myrmarachne* sp. [Pahang]	d162	EU815507*	KM033124	KM033083	KM032992	JX145837*		EU815616*	EU815565*
*Neon reticulatus* (Blackwall, 1853)	d283	KM033191	KM033125	KM033084	KM032993	KM032956			
*Nungia epigynalis* Zabka, 1985	d221	KM033192							KM032924
*Simaetha* sp.	d027	EU815477*	KM033126	KM033085		JX145839*		EU815592*	EU815546*
*Trite pennata* Simon, 1885	d035	EU815478*		KM033086		KM032957	KM033207	EU815593*	EU815547*
**Baviines**									
*Bavia* aff. *aericeps* Simon, 1877 [Sabah]	d079	EU815490*	KM033127			KM032958		EU815603*	KM032925
*Stagetilus* sp. [Selangor]	MRB079	KM033193		KM033087		KM032959			KM032926
**Marpissoids**									
*Afromarengo* sp. [Gabon]	MRB262	JX145758*	KM033128	KM033088	KM032994	JX145842*		JX145682*	JX145905*
*Dendryphantes hastatus* (Clerck, 1757)	d043	EF201646*	KM033129	KM033089				KM033228	KM032927
*Platycryptus californicus* (Peckham & Peckham, 1888)	d316	KM033194		KM033090	KM032995	KM032960		KM033229	
*Rhene* sp. [Pahang]	LiD-001-021-05	EF419044*	EF419010*				EF419134*	EF419108*	EF418979 / EF419160*
*Tisaniba mulu* Zhang & Maddison, 2014	SWK12-1244	KM032876*							KM032880*
**Saltafresians**									
*Aelurillus* cf. *ater* (Kroneberg, 1875)	d140	EU815504*	EU815536*	KM033037	KM032972	JX145831*	KM033199	EU815615*	EU815564*
*Amphidraus complexus* Zhang & Maddison, 2012	JXZ035	KC615380*		KM033038		KC616069*		KC615640*	KC615806*
*Athamas* cf. *whitmeei* O. P.-Cambridge, 1877	JXZ345					KC616286*		KC615649*	KC615822*
*Bacelarella pavida* Szüts & Jocqué, 2001	d195	EU815511*	EU815538*	KM033039	KM032973	KM032946		EU815618*	EU815569*
*Bathippus macrognathus* (Thorell, 1881)	JXZ372	KC615407*		KM033040		KC616305*			KC615835*
*Bianor maculatus* (Keyserling, 1883)	d017	EU815469*		KM033041			KM033200	EU815585*	EU815542*
*Bristowia afra* Szüts, 2004	JXZ363	KC615409*				KC616301*			
*Bristowia afra* Szüts, 2004	MRB230			KM033042				KM033218	
*Cheliceroides longipalpis* Zabka, 1985	d222		KM033111	KM033043		JX145830*		KM033219	EU815579*
*Cheliceroides* cf. *longipalpis* Zabka, 1985	d415	KM033179							
*Chinattus parvulus* (Banks, 1895)	d009	EU815464*	EU815525*	KM033044		JX145848*	KM033201	EU815581*	
*Chinophrys pengi* Zhang & Maddison, 2012	JXZ145	KC615416*		KM033045		KC616146*			KC615843*
*Corythalia locuples* (Simon, 1888)	JXZ315	KC615390*		KM033046		KC616260*		KC615645*	KC615816*
*Cosmophasis umbratica* Simon, 1903	Su et al. 2007	EF419020*					EF419117*	EF419085*	EF418960 / EF419141*
*Cytaea nimbata* (Thorell, 1881)	JXZ229	KC615474*		KM033047		KC616197*		KC615693*	KC615899*
*Diolenius varicus* Gardzińska & Zabka, 2006	JXZ349	KC615480*		KM033048		KC616290*		KC615695*	KC615905*
*Diplocanthopoda marina* Abraham, 1925	d209	KM033180				KM032947		KM033220	KM032911
*Eburneana* sp. [Gabon]	MRB231	KM033181		KM033049		JX145858*		KM033221	KM032912
*Echeclus* sp. [Selangor]	MRB089	KM033182				KM032948		KM033222	KM032913
*Euophrys frontalis* (Walckenaer, 1802)	JXZ137	KC615536*		KM033050		KC616139*			KC615960*
*Evarcha proszynskii* Marusik & Logunov, 1998	d096	DQ665765*	KM033112			EU522704*			DQ665723*
*Evarcha proszynskii* Marusik & Logunov, 1998	d323			KM033051	KM032974				
*Freya decorata* (C. L. Koch, 1846)	d211	EU815521*	EU815539*		KM032975	EU522705*			JX145908*
*Gedea* cf. *tibialis* Zabka, 1985	MRB090	KM033183				KM032949		KM033223	KM032914
*Habrocestum* cf. *albimanum* Simon, 1901	d132	EU815500*						EU815611*	EU815562*
*Habronattus borealis* (Banks, 1895)	d207	KM033184		KM033052	KM032976	KM032950		KM033224	KM032915
*Hasarius adansoni* (Audouin, 1826)	d295		KM033113	KM033053	KM032977				
*Hasarius adansoni* (Audouin, 1826)	S130/S131/S324	AY297281*						AY297409*	
*Heliophanus cupreus* (Walckenaer, 1802)	d044	DQ665769*	KM033114			EU522710*	DQ665710*	DQ665756*	KM032916
*Idastrandia* cf. *orientalis* (Szombathy, 1915)	d108	EU815535; EU815496*	EU815535*			JX145852*		EU815608*	EU815560*
*Langerra* aff. *longicymbium* Song & Chai, 1991	d182	KM033185		KM033054					KM032917
*Leptorchestes berolinensis* (C. L. Koch, 1846)	d086	EU815491*	EU815534*	KM033055				EU815604*	EU815556*
*Longarenus brachycephalus* Simon, 1903	MRB258	JX145798*		KM033056	KM032978	KM032951		JX145707*	KM032918
*Nannenus* sp. [Pahang]	d105	EU815493*		KM033057	KM032979	JX145853*			EU815558*
*Naphrys pulex* (Hentz, 1846)	JXZ081	JX145760*	KM033115		KM032980	JX145844*		KC615749*	JX145907*
*Omoedus orbiculatus* (Keyserling, 1881)	d008							KC615792*	
*Omoedus orbiculatus* (Keyserling, 1881)	JXZ136	JX145762*	KM033116	KM033058		JX145846*	KM033202		
*Omoedus papuanus* Zhang & Maddison, 2012	JXZ286	KC615619*		KM033059		KC616234*		KC615790*	KC616042*
*Pellenes peninsularis* Emerton, 1925	d057	DQ665774*	KM033117	KM033060		JX145864*	DQ665712*		
*Pellenes peninsularis* Emerton, 1925	d400				KM032981				
*Phaulostylus grammicus* Simon, 1902	d304	KM033186		KM033061					
*Philaeus chrysops* (Poda, 1761)	d025	EU815475*	EU815530*	KM033062		JX145855*		EU815590*	EU815545*
*Phintella* sp. [Gabon]	d402	KM033187		KM033063	KM032982				
*Plexippus paykulli* (Audouin, 1826)	LiD-001-029-05		EF419002*				EF419127*		
*Plexippus paykulli* (Audouin, 1826)	MRB016	JX145784*		KM033064		EU522713*			
*Plexippus paykulli* (Audouin, 1826)	S73							AY297384*	AY296674 / AY297317*
*Pochyta* cf. *pannosa* Simon, 1903	MRB257	JX145806*		KM033065	KM032983	KM032952		JX145715*	KM032919
*Saitis barbipes* (Simon, 1868)	JXZ147	KC615589*		KM033066		KC616147*		KC615767*	KC616011*
*Salticus scenicus* (Clerck, 1757)	d003	DQ665777*	KM033118	KM033067	KM032984	EU522719*	DQ665713*	JX145663*	AY296707 / AY297352*
*Thiania bhamoensis* Thorell, 1887	LiD-001-028-05	EF419049 / EF419080*	EF419015*				EF419138*	EF419113*	EF418984 / EF419165*
*Trydarssus* cf. *nobilitatus* (Nicolet, 1849)	MRB270	KM033188		KM033068	KM032985	JX145847*		KM033225	KM032920
*Tusitala lyrata* (Simon, 1903)	MRB226	JX145771*		KM033069		JX145856*		JX145689*	JX145912*
*Yllenus arenarius* Menge, 1868	d013		EU815527*					EU815583*	EU815541*
*Yllenus arenarius* Menge, 1868	JXZ173	JX145766*		KM033070	KM032986	JX145851*			
*Zabkattus furcatus* Zhang & Maddison, 2012	JXZ218	KC615503*		KM033071		KC616190*			KC615928*

Our sample targeted especially the non-salticoid salticids, those that lie outside the major clade of familiar salticids (Maddison and Needham 2006). We included most available data from non-salticoid salticids, both new data and data previously published by Su et al. (2007) and others (Maddison and Hedin 2003, Maddison and Needham 2006, Maddison et al. 2007, Bodner and Maddison 2012, Ruiz and Maddison 2012, Zhang and Maddison 2013, Maddison and Piascik, in press). Included for the first time in a molecular phylogeny are the cocalodines (Wanless 1982, Maddison 2009), which are Australasian non-salticoid salticids. Also analyzed for the first time are the lyssomanine genera *Chinoscopus* and *Pandisus*, the lapsiine *Lapsias*, and the spartaeines *Brettus*, *Meleon*, *Sparbambus*, and *Taraxella*.

Some previously-published data from non-salticoid salticids was either excluded or represented under a different species name here. Excluded are sequences of *Hispo* cf. *frenata*, because its limited data made it unstable in the analyses (see Maddison and Piascik 2014), “*Portia labiata*” from Su et al. (2007), because its identification is in doubt and no voucher specimen is available, and the actin 5C sequence of *Tomomingi* sp. voucher d243, which we discovered to have been a contaminant from the euophryine *Ilargus*. The species labeled as *Phaeacius yixin* by Su et al. (2007) is included here as “*Phaeacius* sp. [Hainan]”, because the specimen was a juvenile female and thus identified with doubt; by its DNA we suspect it is *Phaeacius lancearius*. The specimen labeled as *Mintonia ramipalpis* by Su et al. (2007) is actually a female *Mintonia silvicola*. This misidentification arose because of an error in male-female matching by Wanless (1984), whose female “*Mintonia ramipalpis*” is actually the female of *Mintonia silvicola*. The correct match of male and female *Mintonia silvicola* is evident by intimate co-collecting in a recent expedition to Sarawak (Maddison and Piascik, unpublished) and in DNA sequence comparison. We have therefore blended data from Su et al.’s female with that from our males to represent *Mintonia silvicola*.

**Figures 1–13.**
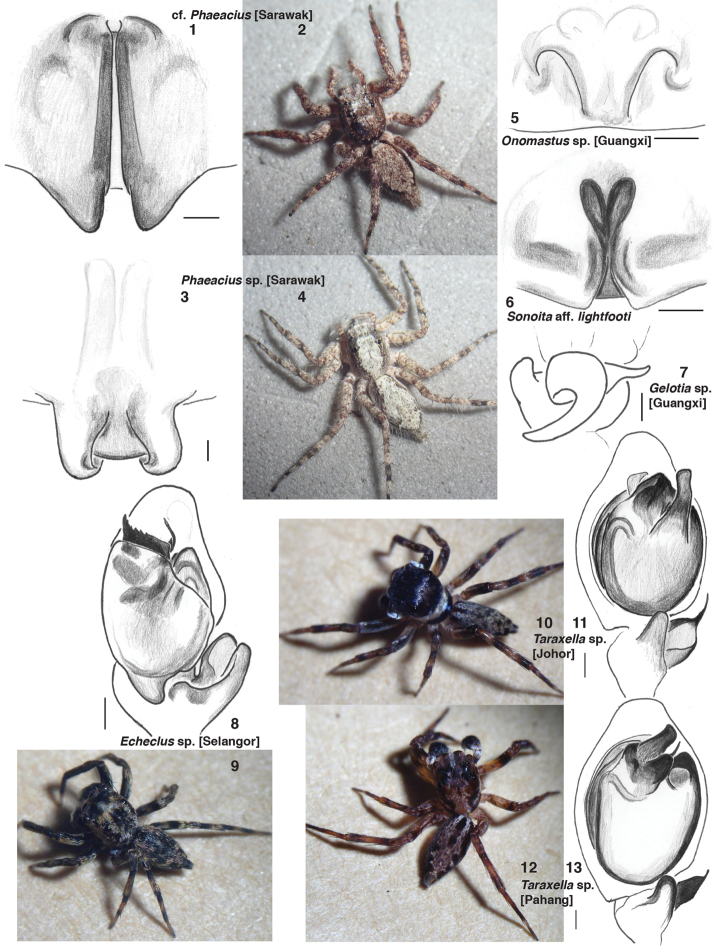
Specimens of undescribed species. **1, 3, 5, 6** are of epigyna, ventral view; **8, 11, 13** of left palps, ventral view; **7** of the right palp tibia, retrolateral view. Scale bar 0.1 mm. **1–2** Female cf. *Phaeacius* [Sarawak], voucher SWK12–3728 **3–4** Female *Phaeacius* sp. [Sarawak], voucher SWK12–4541 **5** female *Onomastus* sp. [Guangxi], voucher MRB085 **6** Female *Sonoita* aff. *lightfooti*, voucher MRB200. **7** male *Gelotia* sp. [Guangxi], voucher MRB199. The drawing is reversed so as to appear to be the left palpus **8–9** Male *Echeclus* sp. [Selangor], voucher MRB089 **10–11** Male *Taraxella* sp.[Johor], voucher d246 for the palpus. The photo of the living male may or may not be of the same specimen **12–13** Male *Taraxella* sp. [Pahang], voucher d248. The photo of the living male may or may not be of the same specimen. Figures **1–13** are copyright ©2014 W.P. Maddison, released under a Creative Commons Attribution (CC-BY) 3.0 license.

Some of the species studied appear to be undescribed, or are doubtfully the same as described species. Following the usual convention, the names of some of our specimens includes “cf.” to indicate that they may be the same as the mentioned species, “aff.” to indicate that they are close to, but distinctly different from, the mentioned species. Figures 1–13 give illustrations of some of the undescribed species, in order to facilitate future association of our data with a species name. The species we refer to as “cf. *Phaeacius* [Sarawak]” (Figs 1, 2) is known from a single female and juvenile from Lambir Hills, Sarawak. It resembles *Phaeacius* but the legs are shorter, and the epigynum is distinctively different. *Phaeacius* sp. [Sarawak] (Figs 3, 4) is a fairly typical *Phaeacius* whose epigynum resembles that of *Phaeacius leytensis* Wijesinghe, 1991, but with the atria elongated posteriorly. *Onomastus* sp. [Guangxi] is shown in Fig. 5. *Sonoita* aff. *lightfooti* (Fig. 6) has longer grooves for the openings of the epigynum than *Sonoita lightfooti*, and is distinctive in gene sequences as well. *Gelotia* sp. [Guangxi] (Fig. 7) has a palp resembling *Gelotia syringopalpis*, but the tibial apophyses are much shorter. *Echeclus* sp. [Selangor] (Figs 8, 9) was identified as an *Echeclus* by the distinctive form of the palp tibia, and the embolus hidden behind a ledge of the tegulum, through which several dark sclerites can be seen (Prószyński 1987). It might equally well have been identified, by the same features, as a *Curubis* species (Zabka 1988). Indeed, the two genera are likely synonyms. “*Echeclus*” is used as that is the older name. *Taraxella* sp. [Johor] (Figs 10, 11) and *Taraxella* sp. [Pahang] (Figs 12, 13) are typical species of *Taraxella*. The specimen MRB024 identified as *Cotinusa* sp. is the same as that named “unidentified thiodinine” by Bodner and Maddison (2012). The *Hypaeus* specimen (d130) was formerly identified as *Acragas* sp. (Bodner and Maddison 2012). The specimen d105 labeled as “*Nannenus lyriger*” by Maddison et al. (2008) is not *Nannenus lyriger*, but another apparently undescribed species of *Nannenus*. The data for *Cheliceroides longipalpis* comes from two specimens, d222 which is clearly *Cheliceroides longipalpis*, and d415 which may be a different but very closely related species. Notes on the undescribed hisponines are given by Maddison and Piascik (2014), whose data we use.

Specimens whose voucher ID’s (Table 1, Suppl. material 1) are of the form S###, d###, MRB###, or JXZ###, SWK12-####, or ECU11-####, where # is a digit, are deposited in the Spencer Entomological Collection of the Beaty Biodiversity Museum, University of British Columbia. The remaining vouchers are in the Lee Kong Chian Natural History Museum (formerly Raffles Museum for Biodiversity Research or RMBR), National University of Singapore.

In addition to analyses done on all 176 sampled taxa (“Complete”), subsets of taxa were analyzed alone. A first subset (“Salticoida”) of 78 taxa highlighted the Salticoida, with just 7 non-salticoid outgroup taxa (4 hisponines, 1 spartaeine, 1 cocalodine, 1 lapsiine), in order to obtain an alignment that was less perturbed by highly divergent non-salticoids. A second subset highlighted the non-salticoids (“Non-salticoid”, 120 taxa), to obtain an alignment primarily for non-salticoid salticids, and also to be able to explore their relationships in more detail.

### Gene choice and sequencing

Eight genes were used for this analysis. Two are nuclear ribosomal genes, 28s and 18s (Maddison and Hedin 2003, Maddison et al. 2008). Four are nuclear protein coding genes: actin 5C (Vink et al. 2008, Bodner and Maddison 2012), *wingless* (Blackledge et al. 2009), myosin heavy chain (“myosin HC”, Blackledge and Hayashi, unpublished), and histone 3 (Su et al. 2007). Two mitochondrial regions were also used, CO1 and another region including 16s and NADH1 ("16sND1", Hedin and Maddison 2001, Maddison and Hedin 2003). Following Bodner and Maddison (2012), the intron region of actin 5C was deleted from the analyses as it is highly variable and difficult to align.

The sequencing protocols for *wingless* and myosin HC are described below. For other genes, sequences marked “^S^” in Table 1 and Suppl. material 1 were obtained by the protocols of Su et al. (2007), all others by the protocols of Bodner and Maddison (2012) and Zhang and Maddison (2013).

For most *wingless* sequences, the forward and reverse primers used were respectively Spwgf1 and Spwgr1 (Blackledge et al. 2009). PCR amplification included a 2 min 94 °C denaturation and 35 cycles of 30 s at 94 °C, a 30 s annealing step at 52–57 °C, 30 s at 72 °C and one 3 min extension step at 72 °C. For some specimens this did not succeed in amplifying *wingless*, and for those we used a nested protocol starting with outer primers wg550F and wgABRz (Wild and Maddison 2008). The resulting product was then amplified using two internal primers, forward Wnt8MBf1 5’-TGTGCTACTCARACKTGYTGG-3’ and reverse Wnt8MBr3 5’-ACAAWGTTCTGCA ACTCATRCG-3’. For both the external and internal reactions amplification was done with 2 min 94 °C denaturation and 37 cycles of 20 s at 94 °C, a 20 s annealing step at 52 °C (wg550F/wgABRz) or 56 °C (wnt8MBf1/wnt8MBr3), and 2 min at 72 °C, and no final extension. The nested protocol obtained sequences for *Bavia* aff. *aericeps* (voucher d389), *Hasarius adansoni* (d295), *Philodromus* sp. (GR011), *Simaetha* sp. (d027), and *Yllenus arenarius* (JXZ173). In other specimens, the nested protocol often resulted in amplification of a different member of the *wingless* family (e.g. WNT-8), but these were readily detected (and excluded) by BLASTing them to other genes in the NCBI database (http://www.ncbi.nlm.nih.gov).

The region of myosin HC sequenced corresponds mostly to an intron. Primers used are (forward) Myhc1f 5'-ACAACAATTCTTCAACCATCAC-3' and (reverse) Myhc5r 5'-CTTCCTCAAGGATGGACA-3' (Blackledge and Hayashi, unpublished). PCR amplification included a 2 min 95 °C denaturation and 35 cycles of 20–45 s at 95 °C, a 45 s annealing step at 52 °C, 1 min at 72 °C and one 10 min extension step at 72 °C. The boundary between the exon and intron was determined by aligning the salticid implicit amino acid translations against the known transcript for myosin HC in *Cyrtophora citricola* (Genbank accession AAM97635.1; Ruiz-Trillo et al. 2002).

Two small single-nucleotide errors in the sequences were corrected after the analyses but before submission to Genbank. These are near the ends of CO1 of MRB199 (*Gelotia* sp. [Guangxi]) and MRB231 (*Eburneana* sp. [Gabon]). Given that CO1 had little resolution, these are unlikely to have affected the results.

### Sequence alignment

Automatic multiple sequence alignment was performed by MAFFT (Katoh et al. 2002, 2005), run via the align package of Mesquite (prerelease of version 3, Maddison and Maddison 2014), aided by Mesquite for manual corrections and for alignment by amino acid. Coding regions were easily aligned by hand according to amino acid translations. This was done starting with an initial automated nucleotide alignment, followed by hand correction in Mesquite using the Color Nucleotide By Amino Acid function to reveal amino acid translation. Non-coding regions (28s, 18s, noncoding region of 16sND1, myosin HC intron) were aligned by MAFFT using the L-INS-i option (--localpair --maxiterate 1000). Mesquite was used to color the matrix via the option ‘‘Highlight Apparently Slightly Misaligned Regions’’ so as to identify regions that needed correction. These were almost always near the ends of sequences.

Alignment was done separately on the Complete, Non-salticoid and Salticoida datasets. Following the MAFFT alignment, the Salticoida dataset required 5 small realignments by hand in 18s. The first 60 positions in the initial alignment of 16s were also realigned locally, and in addition 8 minor shifts by one or two positions were made by hand. The Non-salticoid dataset required three simple hand fixes in 28s. The first 24 positions of 16s in the initial alignment were realigned by MAFFT in isolation because of several obvious misalignments. The Complete dataset appeared poorly aligned in 28s from sites 375 to 489 in the initial alignment, which were therefore realigned by MAFFT in isolation. The first 60 positions in the initial alignment of 16s were also realigned locally, and in addition 8 minor shifts by one or two positions were made by hand. Five small shifts were performed by hand for 18s. Many analyses were done with different variants of the alignments as this study was progressing, and the phylogenetic trees remained substantially consistent.

### Phylogenetic analysis

Phylogenetic analyses using maximum likelihood were run using RAxML version 7.2.8alpha (Stamatakis 2006a, 2006b). The protein coding genes and 16sND1 were each divided into partitions. Protein coding regions were divided into one partition for 1st and 2nd codon positions, and another partition for third codon positions. Introns and non-coding regions were treated as separate partitions. For the fused 8 gene analyses, there were 7 partitions total: (1) 1st + 2nd codon positions in nuclear genes, (2) 3rd codon position nuclear, (3) nuclear intron, (4) nuclear ribosomal, (5) 1st + 2nd codon positions mitochondrial, (6) 3rd codon position mitochondrial, (7) noncoding mitochondrial. Each partition was permitted to have its own model parameters.

Analyses were done for each gene region separately with the Complete taxon set. In addition, analyses fusing all 8 genes were done for the Non-salticoid and Salticoida taxon sets. For all of these, RAxML runs assuming the GTRCAT model were used with 100 search replicates, to seek maximum likelihood trees. In addition, likelihood bootstrap analysis was performed with 500-1500 bootstrap replicates (as indicated in the figures), each involving a single search replicate. Phylogenetic analyses using GARLI version 1.0.699 (Zwickl 2006) under the model GTR+gamma+I were also done but are not reported; they resulted in substantially similar trees.

## Data resources

The data underpinning the analyses reported in this paper are deposited in the Dryad Data Repository at doi: 10.5061/dryad.v53h1.

## Results

Sequences obtained and used in analyses are indicated in Table 1 and Suppl. material 1, along with those sequences taken from the literature.

Figure 14 summarizes the results of the phylogenetic analyses, which are given in more detail in Figures 15–27. Colors assigned to clades in Figure 14 are shown in the remaining figures. Figures 15–19 show the All Genes results for the Complete, Non-salticoid and Salticoida datasets. Figures 20–27 show the results for individual genes analyzed separately.

**Figure 14.**
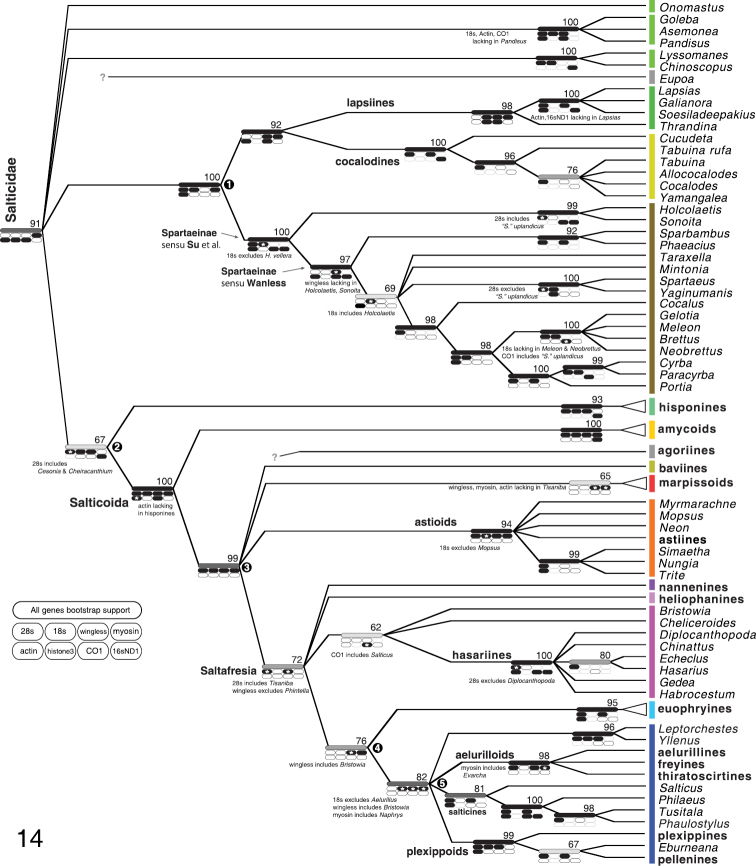
Summary of phylogenetic results. Number above branch shows percentage of maximum likelihood bootstrap replicates with clade. For clades outside the Salticoida, these percentages come from the Non-salticoid dataset with 1500 replicates; within the Salticoida, these come from the Salticoida dataset with 1000 replicates; the Salticoida percentage comes from the Complete dataset with 1000 replicates. Long bar on branch shows same percentage graphically: black 91–100%; dark gray 81–90%; gray 71–80%; light gray 51–70%. Oval spots show presence of clade in maximum likelihood tree for individual genes, with exceptions noted by * and adjacent notes. The notes about *wingless* on the Spartaeinae node and actin on the Salticoida node are ambiguous in placement; they could equally well have been placed one node deeper because of missing data. Pale gray outline indicates no conclusion because of inadequate taxon sampling. All indications of support are from analyses excluding *Eupoa*, agoriines, *Spartaeus spinimanus* and “*Spartaeus*” *uplandicus*. Bars show colors used to highlight taxa in Figs 15–27.

**Figure 15.**
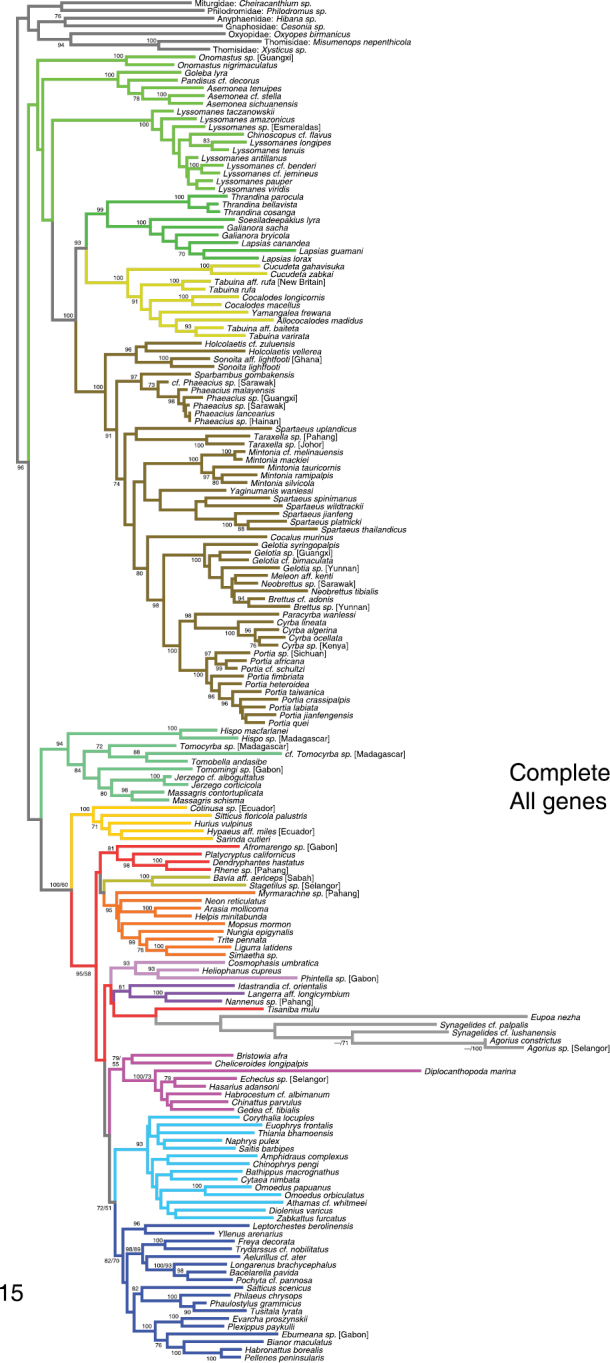
Phylogeny from complete taxon sample, All Genes analysis. Numbers beside branches show percentage of 1000 RAxML likelihood bootstrap replicates with clade in analysis with *Eupoa* and agoriines excluded. In analyses with these taxa included (500 bootstrap replicates), bootstrap percentages are within 5 of those shown, except for branches with two values (e.g. “100/60”), in which case the first value is from an analysis with *Eupoa* and agoriines excluded, the second value with them included. Colors of branches are the same as those highlighting taxa in Fig. 14.

**Figures 16–17.**
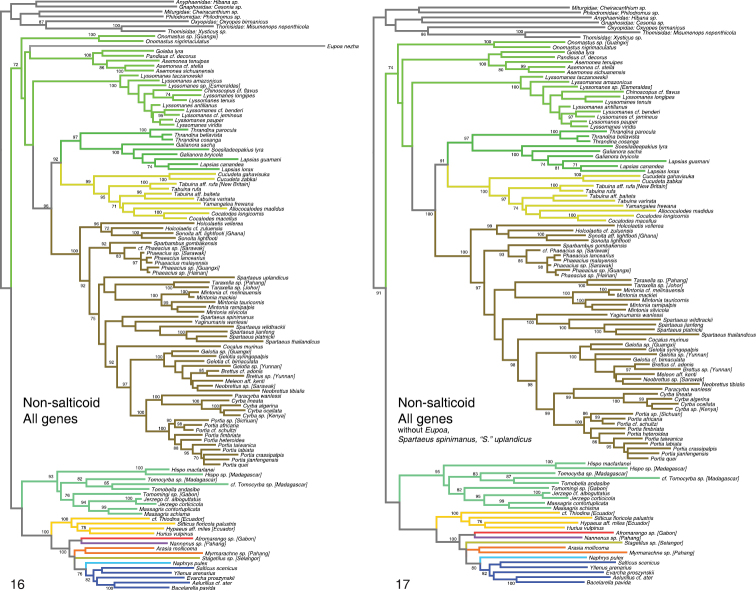
Phylogeny from Non-salticoid dataset, All Genes analysis. Numbers beside branches show percentage of RAxML likelihood bootstrap replicates with clade. **16** Non-salticoid analysis with all taxa included (1500 bootstrap replicates used) **17** Non-salticoid analysis with *Eupoa*, *Spartaeus spinimanus*, and “*Spartaeus*” *uplandicus* excluded (500 bootstrap replicates used). Colors of branches are the same as those highlighting taxa in Fig. 14.

**Figures 18–19.**
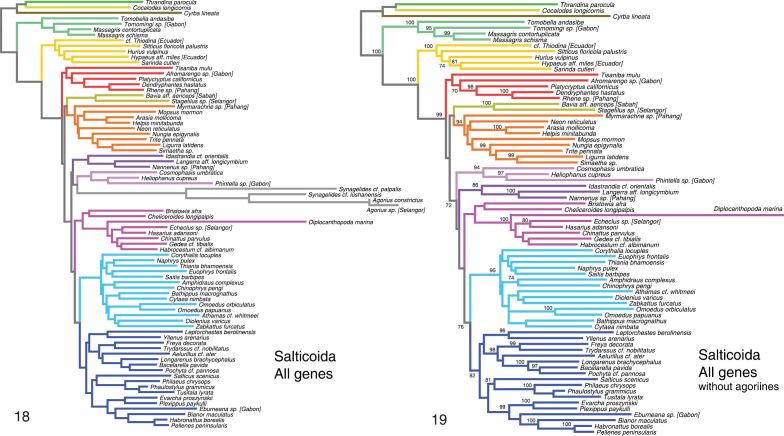
Phylogeny from Salticoida dataset, All Genes analysis. **18** Salticoida analysis with all taxa included **19** Salticoida analysis with *Agorius* and *Synagelides* excluded. Numbers beside branches show percentage of 1000 RAxML likelihood bootstrap replicates with clade. Colors of branches are the same as those highlighting taxa in Fig. 14.

**Figures 20–22.**
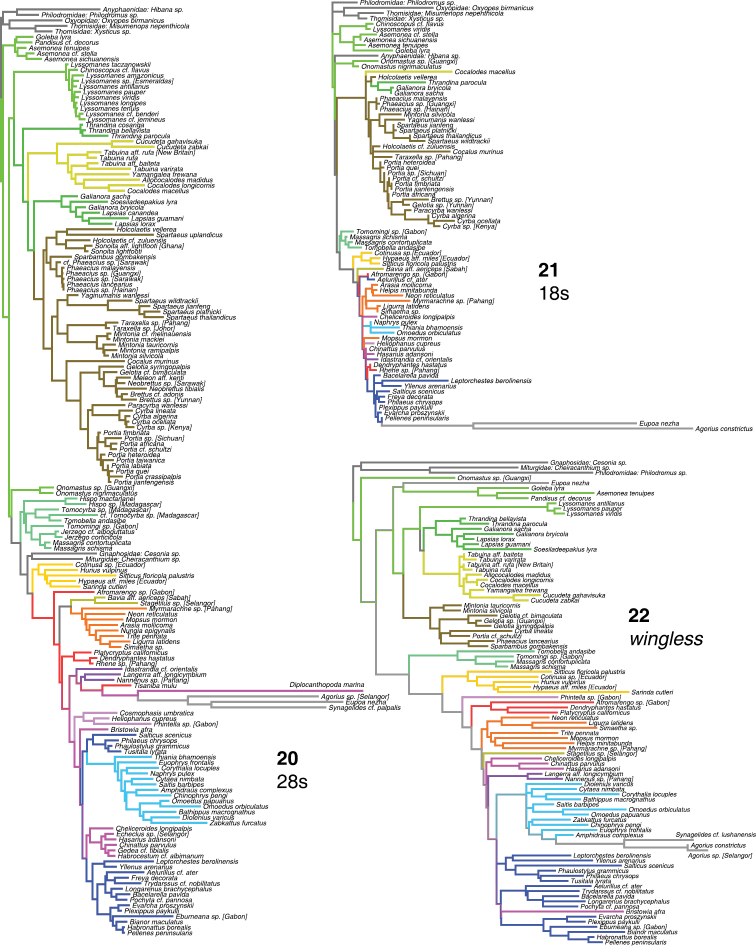
Phylogeny from gene regions analyzed alone, complete taxon sample. **20** 28s **21** 18s **22** *wingless*. Colors of branches are the same as those highlighting taxa in Fig. 14.

**Figures 23–27.**
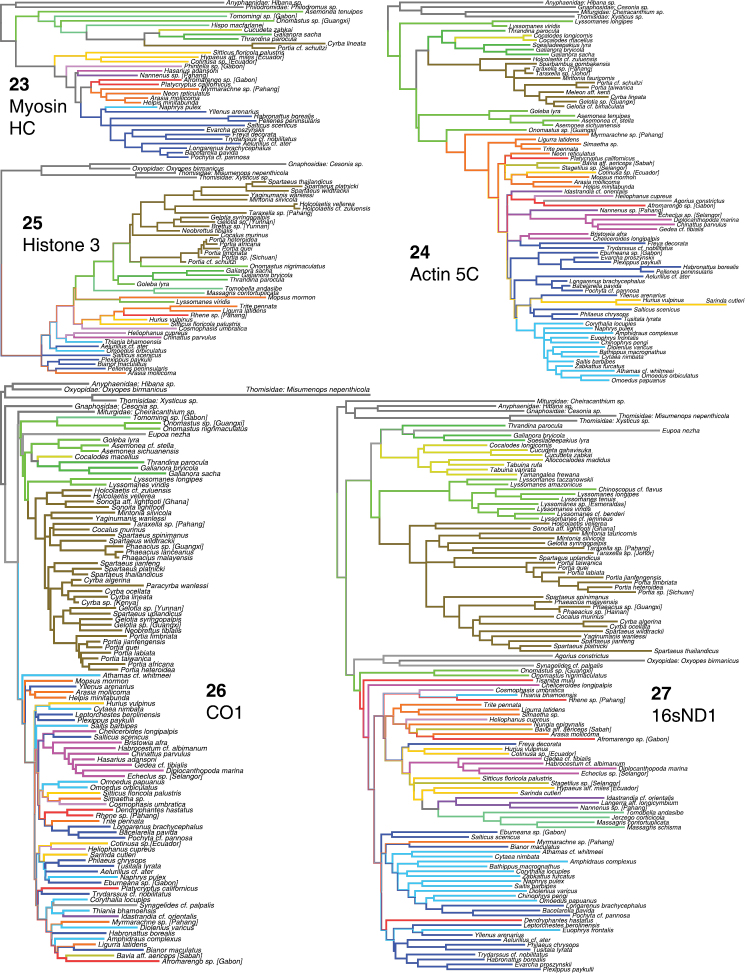
Phylogeny from gene regions analyzed alone, complete taxon sample. **23** myosin HC **24** actin 5C **25** Histone 3 **26** CO1 **27** 16sND1. Colors of branches are the same as those highlighting taxa in Fig. 14.

Several taxa stood out as being problematical, especially for nuclear ribosomal genes. *Eupoa* was not only difficult to sequence (Maddison et al. 2007) but its 28s and 18s genes stand as outliers in alignments, remarkably different from other salticids. The same holds for the agoriines *Agorius* and *Synagelides* and, in 28s, for the hasariine *Diplocanthopoda*. These sequences do not appear to be contaminants, as they BLAST in the NCBI database to salticids. In analyses with just 28s or 18s, these taxa tend to appear on long branches, wandering to different parts of the salticid phylogeny in different analyses, attaching themselves together and to clearly inappropriate relatives (e.g. within the pellenines, Fig. 21). This instability and unexpected placement are likely artifacts due to long branch attraction (Felsenstein 1978), possibly related to compositional bias (Hasegawa and Hashimoto 1993). *Eupoa* and the agoriines have the highest GC bias of the sample (0.72–0.78, compared to 0.60–0.69 for all other species) in 28s, and are similar outliers in 18s. With *wingless*, *Eupoa* appears on a normal-length branch (Fig. 22). However, the agoriines with *wingless* are on a long branch in an unlikely place, within the euophryines (Fig. 22). Their placement is unstable: in slight variants of the analyses they come out in other places. There is nothing obviously unusual about the *wingless* sequences in agoriines, but whatever has shifted the GC bias in the nuclear ribosomal genes might also be affecting the rest of the genome. When *Eupoa* and the agoriines are excluded from analyses, bootstrap percentages rise through much of the tree, suggesting their instability is adding noise to the other relationships in the tree. For this reason, the reported bootstrap percentages and other indications of support are generally those for analysis with *Eupoa* and the agoriines excluded. *Diplocanthopoda* was left in the bootstrap analyses, because CO1, actin 5C and 16sND1 all agree on a clear placement in the hasariines.

## Discussion

Many of the salticid clades now recognized by molecular data had been previously recognized by morphological data. For instance, Wanless (1980, 1984, 1985) recognized the three distinct lyssomanine groups and the Spartaeinae. The Salticoida was strongly supported by many morphological characters (Maddison 1988, 1996, Maddison and Hedin 2003), except that the status of the hisponines was unclear. Wanless (1981) implicitly included the hisponines within the salticoids, while Maddison (1996) did not consider the hisponines in his listing of salticoid synapomorphies. Other groups whose previous formulation by morphology mostly or entirely matches their current boundaries by molecular data are the marpissines (Barnes 1958), euophryines (Prószyński 1976), amycines (Galiano 1968), heliophanines (Maddison 1987), dendryphantines (Maddison 1996), and plexippines (Maddison 1988). At the finer scale, morphological systematics gave us concepts for many genera that are concordant with more recent data.

However, the first molecular data for salticid phylogeny as a whole (Maddison and Hedin 2003) uncovered several unanticipated groups, including the Amycoida, Plexippoida, and Marpissoida. Further data revealed the Astioida and Aelurilloida (Maddison et al. 2008), and later the Saltafresia (Bodner and Maddison 2012). These are major groups within the Salticoida, each uniting several subfamilies.

### Deepest relationships

Our results help resolve or add strength to relationships at the deepest level of salticid phylogeny. Wanless (1980) recognized three major subdivisions of lyssomanines: (1) the New World genera *Lyssomanes* and *Chinoscopus*, (2) the Asian *Onomastus*, and (3) the remaining Old World genera including *Asemonea*. He suggested these three groups are so distinct that they may not belong together. The molecular data agree: the three groups’ divisions are so deep that their relationships have not yet been recovered, and it is possible, even likely, that they do not form a monophyletic group. Different analyses give different results of the relationships of these three, with some showing the New World genera as sister to the spartaeine-lapsiine-cocalodine clade (as recovered by Su et al. 2007), other results showing *Onomastus* in that role, and others showing the three lyssomanine groups together.

Spartaeines, lapsiines and cocalodines form a clade (node 1, Fig. 14). Although Rodrigo and Jackson (1992) concluded that spartaeines, *Holcolaetis* and the *Cocalodes* group form a clade (they were unaware of lapsiines), our analysis provides the first support for such an arrangement – their analysis included only a single taxon outside the group, and therefore it could not speak to the monophyly of the group. Our new result is intuitively appealing, as it groups together all of the extant medium-sized generalized non-salticoids/non-hisponines that are typically brown or gray. However, these presumably are or could be plesiomorphic traits; there had been no obvious reason to expect the spartaeines, lapsiines and cocalodines should have fallen together. There is no known morphological synapomorphy of this clade.

Within this spartaeine-lapsiine-cocalodine clade, the subclade historically best known by morphology is Wanless’s (1984) narrow version of the Spartaeinae, delimited by the presence of a tegular furrow (Wanless 1984). The Spartaeinae sensu stricto is primarily Afro-Eurasian, with a few Australasian species. Outside of this clade, there are no clear morphological synapomorphies defining subclades, and yet there is a striking geographical pattern: all of the Neotropical species belong to a clade, thus forming the lapsiines, while all of the Australasian species belong to a clade, thus forming the cocalodines. It is unsatisfying that we lack morphological synapomorphies for the lapsiines or cocalodines. The data suggest that the lapsiines and cocalodines are sister groups, with spartaeines more distant (Fig. 14).

Our results continue to support the relationship of hisponines with the Salticoida (node 2, Fig. 14; Figs 15–17; Maddison and Needham 2006, Bodner and Maddison 2012).

The placement of *Eupoa* remains unclear. As noted under Results, the 28s and 18s genes of *Eupoa* may be unreliable phylogenetically, although Maddison et al. 2007 found those genes to place *Eupoa* among non-salticoid salticids. In our results *Eupoa* likewise has no clear placement, except for being outside the clade of Salticoida + Hisponinae. This result appears in the Non-salticoid and Complete datasets, and with the separate analyses of *wingless*, CO1, and 16sND1.

### Spartaeinae

Our results strongly support the monophyly of the Spartaeinae sensu Su et al. (2007), placing *Holcolaetis* and *Sonoita* together with the Spartaeinae in the narrow sense. This is concordant with Wanless’s (1985) hypothesis that *Holcolaetis* and *Sonoita* formed a clade with the spartaeines to the exclusion of *Cocalodes*. The analyses of Su et al. (2007) did not sample *Sparbambus*, *Taraxella*, *Brettus* or *Meleon*, but otherwise their results were largely concordant with ours, which are: (1) *Phaeacius* (with *Sparbambus*) diverge deep, (2) *Yaginumanus* is sister to *Spartaeus*, (3) *Gelotia*, *Neobrettus*, *Brettus* and *Meleon* are monophyletic, (4) *Paracyrba* and *Cyrba* are sisters, (5) *Portia* is sister to *Cyrba* and *Paracyrba*. There is strong support for *Gelotia* through *Cyrba* as a monophyletic group, and for their relationship with *Cocalus*. By our data the exact placements of *Taraxella* and *Mintonia* are unclear.

A few spartaeine taxa in our analyses were problematical in appearing unstable, having different placements by different analyses. One of these is *Spartaeus spinimanus*, for which we have only 16sND1 and CO1 data, both gene regions that appear to evolve too quickly for reliable phylogenetic placement at this level (Bodner and Maddison 2012, Zhang and Maddison 2013). The other is “*Spartaeus*” *uplandicus*, whose 28s sequence appears strongly divergent from others. This sequence is from Maddison and Hedin (2003, as “unidentified spartaeine”, vouchers 185 and 186), and it groups “*Spartaeus*” *uplandicus* with one species of *Holcolaetis*, against the placements by morphology, CO1 and 16sND1. There is a chance that this gene was mis-sequenced in “*Spartaeus*” *uplandicus.* Because of the instability generated, we excluded *Spartaeus spinimanus* and “*Spartaeus*” *uplandicus* from our analyses giving bootstrap results.

Because of the concordance of our phylogenetic results with those of Su et al. (2007), our phylogeny continues to support their conclusions on the stepwise evolution of a complex predatory strategy in spartaeines.

### Deep Salticoid relationships

The Salticoida’s basal divergence places the primarily-Neotropical Amycoida as sister group to an unnamed clade (node 3, Fig. 14) that contains most of salticid diversity. This surprising result, first discovered by Maddison and Hedin (2003), had very strong support in the analyses of Bodner and Maddison (2012). We here add support from two new genes, *wingless* and myosin HC, both of which independently resolve both the Amycoida and its sister group as monophyletic.

There have been hints of a clade uniting the Marpissoida, Astioida and baviines (Bodner and Maddison 2012). In our analyses the clade does not receive bootstrap support above 50% in the Complete or Salticoida analyses. The maximum likelihood trees either show the three as monophyletic or not, depending on taxon inclusion and details of the analysis (e.g., Figs 15 and 18). At present we must conclude the relationship between these three and the Saltafresia is unresolved.

### Astioida

The astioids as delimited by Maddison et al. (2008) continue to be resolved as a clade, with new support from myosin HC and *wingless* (Figs 18, 22, 23). Although the body form of *Nungia* resembles that of baviines and the marpissoid *Metacyrba*, our data clearly place it as an astioid.

### Saltafresia

Bodner and Maddison (2012) proposed a clade, the Saltafresia, containing salticoids other than amycoids, astioids, baviines and marpissoids. They found this clade reasonably well supported – 0.78 likelihood bootstrap and 1.0 posterior probability – but no single gene supported it on its own. Our data here continue to support it when all genes are combined. Two genes support it separately, with the exception of single taxa: 28s (but *Tisaniba* is included) and *wingless* (but *Phintella* is excluded).

### Hasarieae

Previous work had established *Habrocestum* and *Chinattus* as close relatives of *Hasarius* (Maddison et al. 2008). We here add several more genera to the group, all Asian. These are *Gedea*, *Echeclus* and *Diplocanthopoda*. The relationships among these genera are not clearly resolved except for a well-supported relationship between *Hasarius* and *Echeclus* (Figs 14, 19).

### Salticinae

The relationship between *Salticus* and the *Philaeus* group proposed by Maddison et al. (2008) receives additional support from *wingless*, along with previously-demonstrated support from 28s and actin. With high posterior probabilities (Bodner and Maddison 2008) and reasonable likelihood bootstrap values (Figs 15, 19), and supported by different genes independently (Figs 20, 22, 24), this relationship can now be considered sufficiently secure that we here formally place the genera of the *Philaeus* group into a subfamily — the Salticinae. In addition to genera previously analyzed (*Salticus*, *Philaeus*, *Carrhotus*, *Tusitala*, *Mogrus*, and *Pignus*) the subfamily also includes *Phaulostylus*, which is related to *Tusitala* (Fig. 14).

### Plexippoida + Aelurilloida + Leptorchesteae + Salticinae (Node 5)

A set of four major groups (plexippoids, aelurilloids, leptorchestines and the Salticinae) form a clade in our analyses (node 5, Fig. 14). This group is resolved in the All Genes analyses with high bootstrap values, and it appears, almost, in the independent analyses of each of three genes (18s, *wingless*, myosin HC). We say “almost” because three of the genes have one or two taxa missing from or added to the group (Fig. 14). While we believe the evidence is good that these form a clade, there is a possibility that the Euophryinae might also fall nested within it. For instance, in the analyses of Bodner and Maddison (2012) the euophryines were placed as sister to the plexippoids. In our analyses the Euophryinae is placed as sister to the Plexippoida + Aelurilloida + Leptorchesteae + Salticinae.

This major clade is almost entirely Afro-Eurasian, with the plexippoid *Habronattus* being the only exception with more than a handful of species (others are *Pellenes*, *Sibianor*, *Evarcha*, *Phlegra*, *Paramarpissa* and *Salticus*, each with fewer than 15 described New World species).

### Euophryinae

The 14 euophryine taxa in the analyses are resolved strongly as a monophyletic group. This is a stronger test of monophyly than that of Zhang and Maddison (2013), because it includes additional genes and more non-euophryine taxa. The All Genes analyses, along with *wingless* and myosin HC individually, suggest that the euophryines are the sister group to node 5 (Fig. 14).

### Agoriines

Morphologically, the antlike agoriines *Agorius* and *Synagelides* are puzzling, with strangely contorted legs and unusual genitalia (Szüts 2003, Logunov and Hereward 2006, Prószyński 2009). While they appear to be salticoids, morphology has given little guidance as to their placement. As noted already, their 28s and 18s genes appear anomalous, and give no clear indication as to their relationships. In the All Genes analysis their placement is ambiguous, though they appear to be salticoids. In an attempt to determine their placement, an additional analysis was done, using a dataset that included *Agorius constrictus* and a chimera of *Synagelides* cf. *lushanensis* and *Synagelides* cf. *palpalis* (to have a single *Synagelides* taxon with three genes). The aberrant nuclear ribosomal genes of agoriines were excluded from the analysis. The other taxa included were the 70 taxa having at least 4 genes other than CO1 and histone 3. A RAxML likelihood analyses placed *Agorius* and *Synagelides* within the sister group of the Amycoida (node 3, Figure 14) with high support (bootstrap percentage 88), but exactly where was highly unstable. Among the 100 likelihood non-bootstrap search replicates were 7 different placements: sister to leptorchestines, sister to baviines, sister to node 5 in Figure 14, sister to the Saltafresia, sister to astioids + marpissoids + baviines, sister to node 3, or sister to node 3 without the baviines. While a relationship with the leptorchestines is appealing, as it would allow their antlike body forms to be homologous, the best we can say at present is that agoriines likely belong within the sister group of amycoids (node 3).

### Generic limits

Most of the genera for which we have multiple species – e.g., *Asemonea*, *Portia*, *Mintonia*, *Phaeacius*, *Cyrba* – are inferred to be monophyletic in our analyses, corroborating existing concepts based on morphology. The clearest exception is *Tabuina*, in which *Tabuina rufa* and the similar *Tabuina* aff. *rufa* fall apart from the type species *Tabuina varirata*, which had been anticipated as a possibility by Maddison (2009). *Lyssomanes*, *Galianora*, and *Gelotia* are reconstructed as paraphyletic, but in each case the bootstrap values are low.

The placement of cf. *Phaeacius* [Sarawak] as sister to *Phaeacius*, with strong molecular divergence from the other species, would justify establishing a new genus for it.

### Behaviour of individual genes

Previous work (Maddison and Hedin 2003, Bodner and Maddison 2012) has suggested that 28s and actin 5C are phylogenetically informative to a reasonable degree for deeper salticid phylogeny, insofar as their results are concordant with summed genes analyses, morphological resemblances, and biogeographical patterns. 16sND1 is useful at the shallower levels (Hedin and Maddison 2001) but has difficulties recovering deeper relationships, while CO1 struggles through both shallow and deep levels (Maddison and Hedin 2003, Bodner and Maddison 2012).

One surprise in our analyses was the informative behaviour of CO1 in deeper relationships among the non-salticoid salticids. Although CO1 is almost nonsensical in its inferred relationships within the Salticoida, it succeeds in recovering the Spartaeinae, the Spartaeineae sensu Wanless, the lapsiines, and the Salticoida as monophyletic.

Two new genes added, *wingless*, myosin HC, both show clear concordance with the 28s and previous all genes analyses. *Wingless* supports many of the previously recognized clades, including the Salticoida, Amycoida, the sister clade to Amycoida, Plexippoida, Marpissoida (in part), Astioida (in part), Spartaeinae sensu Wanless, and lapsiines. We find it encouraging that a haphazardly chosen protein-coding gene, independent from 28s, supports previous molecular results in Salticidae. There are still, however, many aspects of salticid relationships yet to be resolved, such as the deepest relationships in the family, including the relationships among the three subgroups of lyssomanines, the placement of *Eupoa* and the agoriines, and the relationships among astioids, marpissoids, baviines and the Saltafresia. With the coming era of genomic data, we expect large quantities of new data will be available for exploring these relationships.

## Supplementary material

Supplementary material 1 Specimens used in phylogenetic analyses, with localities and GenBank numbers of sequences indicated. Data type: Occurence; geographic locality; sex. This dataset is made available under the Open Database License (http://opendatacommons.org/licenses/odbl/1.0/). The Open Database License (ODbL) is a license agreement intended to allow users to freely share, modify, and use this Dataset while maintaining this same freedom for others, provided that the original source and author(s) are credited.Wayne Maddison, Daiqin Li, Melissa Bodner, Junxia Zhang, Xu Xin, Qinqing Liu, Fengxiang Liu
