# Supplementary material for: The deep phylogeny of jumping spiders (Araneae, Salticidae)
Source: Zookeys. 2014 Sep 15;(440):57–87. doi: 10.3897/zookeys.440.7891 (PMC4195940; doi:10.3897/zookeys.440.7891)
Supplement: Supplementary material 1 — Specimens used in phylogenetic analyses, with localities and GenBank numbers of sequences indicated. [file zookeys-440-057-s001.docx]

Supplementary material 1

**Table 1.** Specimens and sequences used in phylogenetic analyses, with GenBank numbers indicated. * marks previously published sequences.

|  | Specimen Reference | Sex | Locality | 28s | 18s | *wingless* | myosin HC | actin 5c | histone 3 | CO1 | 16sND1 |
| --- | --- | --- | --- | --- | --- | --- | --- | --- | --- | --- | --- |
| **Outgroups** |  |  |  |  |  |  |  |  |  |  |  |
| Anyphaenidae: *Hibana* sp. | s318 |  | Mexico: Sonora | AY297295* | KM033091 |  | KM032961 | KM032929 |  | AY297422* | AY297295/AY297358* |
| Gnaphosidae: *Cesonia* sp. | s319 | f | Mexico: Sonora: Alamos | AY297293; EF201663* |  | KM032996 |  | EU522700* | DQ665720* | AY297420* | AY296711/AY297356* |
| Miturgidae: *Cheiracanthium* sp. | s321 | m | Mexico: Sonora | AY297294; EF201664* |  | KM032997 |  | KM032928 |  | AY297421* | AY296712/AY297357* |
| Oxyopidae: *Oxyopes birmanicus* Thorell, 1887 | Su et al. 2007 | f | Singapore | EF419032/EF419065* | EF418998* |  |  |  | EF419126* | EF419097* | EF418969/EF419150* |
| Philodromidae: *Philodromus alascensis* Keyserling, 1884 | GR011 |  | Canada: British Columbia | KM033130 | KM033092 | KM032998 | KM032962 |  |  |  |  |
| Thomisidae: *Misumenops nepenthicola* (Pocock, 1898) | Su et al. 2007 | f | Singapore | EF419029/EF419062* | EF418996* |  |  |  | EF419123* | EF419094* | EF418967/EF419148* |
| Thomisidae: *Xysticus* sp. | s316 | m | U.S.A.: Colorado: Monte Vista | AY297296; EF201665* | KM033093 |  |  | EU522701* | DQ665704* | AY297296* | AY296714/AY297359* |
|  |  |  |  |  |  |  |  |  |  |  |  |
| **Lyssomanines** |  |  |  |  |  |  |  |  |  |  |  |
| *Asemonea sichuanensis* Song & Chai, 1992 | SC-03-0055 | f | China: Sichuan: Mt. Ermei |  | EF418986* |  |  |  |  | EF419082* |  |
| *Asemonea sichuanensis* Song & Chai, 1992 | MRB084 | m | China: Guangxi: Ningming County | KM033131 |  |  |  | KM032931 |  |  |  |
| *Asemonea* cf. *stella* Wanless, 1980 | MRB083 | f | South Africa: Kwazulu-Natal Province | JX145767* | KM033094 |  |  | KM032930 |  | JX145686* |  |
| *Asemonea tenuipes* (O. P.-Cambridge, 1869) | d186 | f | Singapore | KM033132 | KM033095 | KM032999 | KM032963 | KM032932 |  |  |  |
| *Chinoscopus* cf. *flavus* (Peckham, Peckham & Wheeler, 1889) | d273 | f | Panama: Gamboa | KM033133 | KM033096 |  |  |  |  |  | KM032888 |
| *Goleba lyra* Maddison & Zhang, 2006 | d051 | m | Madagascar: Fianarantsoa | DQ665768* | KM033097 | KM033000 |  | EU522709* | DQ665707* | DQ665755* |  |
| *Lyssomanes amazonicus* Peckham & Wheeler, 1889 | ECU11-6112 | m | Ecuador: Orellana: Yasuní | KM033134 |  |  |  |  |  |  | KM032889 |
| *Lyssomanes antillanus* Peckham & Wheeler, 1889 | d298 | m | Dominican Republic: El Seibo | KM033135 |  | KM033001 |  |  |  |  |  |
| *Lyssomanes* cf. *benderi* Logunov, 2002 | ECU11-5402 | m | Ecuador: Orellana: Yasuní | KM033136 |  |  |  |  |  |  | KM032890 |
| *Lyssomanes* cf. *jemineus* Peckham & Wheeler, 1889 | ECU11-5682 | m | Ecuador: Orellana: Yasuní | KM033137 |  |  |  |  |  |  | KM032891 |
| *Lyssomanes longipes* (Taczanowski, 1871) | MRB086 | m | French Guiana: Commune of Régina | KM033138 |  |  |  | KM032933 |  | KM033208 | KM032892 |
| *Lyssomanes pauper* Mello-Leitão, 1945 | d297 | m | Uruguay: Lavalleja | KM033139 |  | KM033002 |  |  |  |  |  |
| *Lyssomanes taczanowskii* Galiano, 1980 | ECU11-4193 | f | Ecuador: Orellana: Yasuní | KM033141 |  |  |  |  |  |  | KM032894 |
| *Lyssomanes tenuis* Peckham & Wheeler, 1889 | ECU11-4869 | m | Ecuador: Orellana: Yasuní | KM033142 |  |  |  |  |  |  | KM032895 |
| *Lyssomanes viridis* (Walckenaer, 1837) | s160 |  | U.S.A.: Mississippi | AY297231* |  |  |  |  |  | AY297360* | AY296652/AY297297* |
| *Lyssomanes viridis* (Walckenaer, 1837) | d129 | f | U.S.A.: Mississippi |  | KM033098 | KM033003 |  | EU522715* | DQ665715* |  |  |
| *Lyssomanes* sp. [Esmeraldas] | d408 | j | Ecuador: Esmeraldas: Reserva Canande | KM033140 |  |  |  |  |  |  | KM032893 |
| *Onomastus nigrimaculatus* Zhang & Li, 2005 | Su et al. 2007 | f | China: Yunnan: Xishuangbanna | EF419031/EF419064* | EF418997* |  |  |  | EF419125* | EF419096* | EF418968/EF419149* |
| *Onomastus* sp. [Guangxi] | MRB085 | f | China: Guangxi, Fangchenggang City | JX145768* | KM033099 | KM033004 | KM032964 | KM032934 |  | JX145687* | JX145910* |
| *Pandisus* cf. *decorus* Wanless, 1980 | d303 | f | Madagascar: Toamasina Province | KM033143 |  | KM033005 |  |  |  |  |  |
|  |  |  |  |  |  |  |  |  |  |  |  |
| **Cocalodines** |  |  |  |  |  |  |  |  |  |  |  |
| *Allococalodes madidus* Maddison, 2009 | d236 | m | Papua New Guinea: Southern Highlands Prov. | KM033144 |  | KM033006 |  |  |  |  | KM032896 |
| *Cocalodes longicornis* Wanless, 1982 | d291 | m | Papua New Guinea: Central Province | KM033145 |  | KM033007 |  | KM032935 |  |  | KM032897 |
| *Cocalodes macellus* (Thorell, 1878) | d230 | m | Papua New Guinea: Southern Highlands Prov. | KM033146 | KM033100 | KM033008 |  | KM032936 |  | KM033209 |  |
| *Cucudeta gahavisuka* Maddison, 2009 | d234 | f | Papua New Guinea: Eastern Highlands Prov. | KM033147 |  | KM033009 |  |  |  |  | KM032898 |
| *Cucudeta zabkai* Maddison, 2009 | d235 | f | Papua New Guinea: Southern Highlands Prov. | KM033148 |  | KM033010 | KM032965 |  |  |  | KM032899 |
| *Tabuina* aff. *baiteta* Maddison, 2009 | d313 | f | Papua New Guinea: New Britain | KM033149 |  | KM033011 |  |  |  |  |  |
| *Tabuina rufa* Maddison, 2009 | d232 | m | Papua New Guinea: Enga Province | KM033151 |  | KM033013 |  |  |  |  | KM032900 |
| *Tabuina* aff. *rufa* Maddison, 2009 | d312 | m | Papua New Guinea: New Britain | KM033150 |  | KM033012 |  |  |  |  |  |
| *Tabuina varirata* Maddison, 2009 | d233 | j | Papua New Guinea: Central Province | KM033152 |  | KM033014 |  |  |  |  | KM032901 |
| *Yamangalea frewana* Maddison, 2009 | d231 | m | Papua New Guinea: Eastern Highlands Prov. | KM033153 |  | KM033015 |  |  |  |  | KM032902 |
|  |  |  |  |  |  |  |  |  |  |  |  |
| **Spartaeines** |  |  |  |  |  |  |  |  |  |  |  |
| *Brettus* cf. *adonis* Simon, 1900 | SWK12-4323 | m | Malaysia: Sarawak: Lambir Hills | KM033154 |  |  |  |  |  |  |  |
| *Brettus* sp. [Yunnan] | LiD-026-053-05 | f | China: Yunnan: Mengla County | KM033155 ^S^ | KM033101 ^S^ |  |  |  | KM033195 ^S^ |  |  |
| cf. *Phaeacius* sp. [Sarawak] | SWK12-3728 | f | Malaysia: Sarawak: Lambir Hills | KM033156 |  |  |  |  |  |  |  |
| *Cocalus murinus* Simon, 1899 | LiD-013-027-05 | f | Singapore | EF419019/EF419053* | EF418988* |  |  |  | EF419116* | EF419084* | EF418959/EF419140* |
| *Cyrba algerina* (Lucas, 1846) | Su et al. 2007 | f | Kenya | EF419021/EF419054* | EF418989* |  |  |  |  | EF419086* | EF418961/EF419142* |
| *Cyrba lineata* Wanless, 1984 | MRB106 | f | South Africa: Songimvelo Nature Reserve | JX145792* |  | KM033016 | KM032966 | KM032937 |  | JX145704* |  |
| *Cyrba ocellata* (Kroneberg, 1875) | Su et al. 2007 | f | China: Hainan: Ledong County |  | EF418990* |  |  |  |  | EF419087* | EF418962/EF419143* |
| *Cyrba ocellata* (Kroneberg, 1875) | MRB104 | f | China: Guangxi: Dongxing City | KM033157 |  |  |  |  |  |  |  |
| *Cyrba* sp. [Kenya] | Su et al. 2007 | f | Kenya | EF419023/EF419056* | EF418991* |  |  |  |  | EF419088* |  |
| *Gelotia* cf. *bimaculata* Thorell, 1890 | d250 | j | Malaysia: Pahang | KM033158 |  | KM033017 |  | KM032938 |  |  |  |
| *Gelotia syringopalpis* Wanless, 1984 | Su et al. 2007 | f | China: Hainan: Ledong County | EF419024/EF419057* |  |  |  |  | EF419118* |  |  |
| *Gelotia syringopalpis* Wanless, 1984 | MRB105 | f | China: Guangxi: Ningming County |  |  | KM033019 |  |  |  | KM033212 | KM032903 |
| *Gelotia* sp. [Guangxi] | MRB199 | m | China: Guangxi: Ningming Co. |  |  | KM033018 |  | KM032939 |  | KM033210 |  |
| *Gelotia* sp. [Yunnan] | LiD002-053-05 | f | China: Yunnan: Mengla County |  | KM033102 ^S^ |  |  |  | KM033196 ^S^ | KM033211 ^S^ |  |
| *Holcolaetis vellerea* Simon, 1910 | Su et al. 2007 | f | Kenya | EF419025/EF419058* | EF418992* |  |  |  | EF419119* | EF419090* | EF418963/EF419144* |
| *Holcolaetis* cf. *zuluensis* Lawrence, 1937 | d036 | j | South Africa: Kwazulu-Natal Province | DQ665770* | KM033103 |  |  | EU522711* | DQ665721* | DQ665757* |  |
| *Meleon* aff. *kenti* (Lessert, 1925) | d287 | m | Madagascar: Fianarantsoa | KM033159 |  |  |  | KM032940 |  |  |  |
| *Mintonia mackiei* Wanless, 1984 | SWK12-4202 |  | Malaysia: Sarawak: Lambir Hills | KM033161 |  |  |  |  |  |  |  |
| *Mintonia* cf. *melinauensis* Wanless, 1984 | d441 | m | Malaysia: Sarawak: Kuching | KM033160 |  |  |  |  |  |  |  |
| *Mintonia ramipalpis* (Thorell, 1890) | SWK12-1442 | f | Malaysia: Sarawak: Mulu National Park | KM033162 |  |  |  |  |  |  |  |
| *Mintonia silvicola* Wanless, 1987 | d104 | m | Malaysia: Pahang |  |  | KM033020 |  |  |  |  | KM032904 |
| *Mintonia silvicola* Wanless, 1987 | SWK12-1653 | f | Malaysia: Sarawak: Mulu National Park | KM033163 |  |  |  |  |  |  |  |
| *Mintonia silvicola* Wanless, 1987 | Su et al. 2007 | f | Singapore |  | EF418995* |  |  |  | EF419122* | EF419093* |  |
| *Mintonia tauricornis* Wanless, 1984 | d249 | m | Malaysia: Pahang | KM033164 |  | KM033021 |  | KM032941 |  |  | KM032905 |
| *Neobrettus tibialis* (Prószyński, 1978) | LiD-001-055-05 | f | Malaysia: Pahang: Genting Highlands | EF419030/EF419063* |  |  |  |  | EF419124* | EF419095* |  |
| *Neobrettus* sp. [Sarawak] | SWK12-1040 | f | Malaysia: Sarawak: Mulu National Park | KM033165 |  |  |  |  |  |  |  |
| *Paracyrba wanlessi* Zabka & Kovac, 1996 | Su et al. 2007 | f | Malaysia: Selangor: Ulu Gombak | EF419033/EF419066* | EF418999* |  |  |  |  | EF419098* |  |
| *Phaeacius lancearius* (Thorell, 1895) | d111 | m | Myanmar: Yangon Division | DQ665775* |  | KM033022 |  |  |  | DQ665759* |  |
| *Phaeacius malayensis* Wanless, 1981 | Su et al. 2007 | f | China: Yunnan: Xishuangbanna | EF419034/EF419067* | EF419000* |  |  |  |  | EF419099* | EF418970/EF419151* |
| *Phaeacius* sp. [Guangxi] | LQ-24-06 | f | China: Guangxi: Dongxing | KM033166^S^ | KM033104 ^S^ |  |  |  |  | KM033213 ^S^ | KM032906 ^S^ |
| *Phaeacius* sp. [Hainan] | Su et al. 2007 | f | China: Hainan: Ledong County | EF419035/EF419068* | EF419001* |  |  |  |  |  | EF418971/EF419152* |
| *Phaeacius* sp. [Sarawak] | SWK12-4541 | f | Malaysia: Sarawak: Lambir Hills | KM033167 |  |  |  |  |  |  |  |
| *Portia africana* (Simon, 1886) | Su et al. 2007 | f | Kenya | EF419037/EF419069* | EF419003* |  |  |  | EF419128* | EF419101* |  |
| *Portia crassipalpis* (Peckham & Peckham, 1907) | SWK12-2354 | m | Malaysia: Sarawak: Mulu National Park | KM033168 |  |  |  |  |  |  |  |
| *Portia fimbriata* (Doleschall, 1859) | LiD-001-04 | f | Singapore | EF419038/EF419070* | EF419004* |  |  |  | EF419129* | EF419102* | EF418973/EF419154* |
| *Portia heteroidea* Xie & Yin, 1991 | Su et al. 2007 | f | China: Sichuan: Mt. Ermei | EF419039/EF419071* | EF419005* |  |  |  | EF419130* | EF419103* | EF418974/EF419155* |
| *Portia jianfeng* Song & Zhu, 1998 | Su et al. 2007 | f | China: Hainan: Sanya | EF419040/EF419072* | EF419006* |  |  |  |  | EF419104* | EF418975/EF419156* |
| *Portia labiata* (Thorell, 1887) | S206 |  | Philippines: Luzon | AY297232* |  |  |  |  |  | AY297361* | AY296653/AY297298* |
| *Portia* cf. *schultzi* Karsch, 1878 | d131 | f | Madagascar: Fianarantsoa | DQ665776* | KM033105 | KM033023 | KM032967 | EU522718* | DQ665708* |  |  |
| *Portia quei* Zabka, 1985 | Su et al. 2007 | f | China: Yunnan: Mengla County | EF419042/EF419074* | EF419008* |  |  |  | EF419132* | EF419106* | EF418977/EF419158* |
| *Portia taiwanica* Zhang & Li, 2005 | MRB103 | m | China: Guangxi: Tianlin County | KM033169 |  |  |  | KM032942 |  | KM033214 | KM032907 |
| *Portia* sp. [Sichuan] | SC-03-0011 | f | China: Sichuan: Qingchuan County | EF419043/EF419075* | EF419009* |  |  |  | EF419133* |  | EF418978/EF419159* |
| *Sonoita lightfooti* Peckham & Peckham, 1903 | d226 | f | South Africa: Kwazulu-Natal: Lake St. Lucia | KM033170 |  |  |  |  |  | KM033215 |  |
| *Sonoita* aff. *lightfooti* Peckham & Peckham, 1903 | MRB200 | f | Ghana: N. of Cape Coast, Kakum Forest | JX145791* |  |  |  |  |  | JX145705* | JX145927* |
| *Sparbambus gombakensis* Zhang, Woon & Li, 2006 | d251 | f | Malaysia: Selangor | KM033171 |  | KM033024 |  | KM032943 |  |  |  |
| *Spartaeus jianfengensis* Song & Chai, 1991 | Su et al. 2007 | f | China: Hainan: Sanya | EF419045/EF419076* | EF419011* |  |  |  |  | EF419109* | EF418980/EF419161* |
| *Spartaeus platnicki* Song, Chen & Gong, 1991 | SC-03-069 |  | China: Guizhou: Mt. Longquan | EF419046/EF419077* | EF419012* |  |  |  | EF419135* | EF419110* | EF418981/EF419162* |
| *Spartaeus spinimanus* (Thorell, 1878) | S199 |  | Australia:Queensland |  |  |  |  |  |  | KM033216 | KM032908 |
| *Spartaeus thailandicus* Wanless, 1984 | BV-004 | f | China: Yunnan: Mengla County | EF419047/EF419078* | EF419013* |  |  |  | EF419136* | EF419111* | EF418982/EF419163* |
| *Spartaeus uplandicus* Barrion & Litsinger, 1995 | S185/S186 | f | Philippines: Luzon | AY297233* |  |  |  |  |  | AY297363* | AY296655* |
| *Spartaeus wildtrackii* Wanless, 1987 | Su et al. 2007 | f | Malaysia: Negeri Sembilan | EF419048/EF419079* | EF419014* |  |  |  | EF419137* | EF419112* | EF418983/EF419164* |
| *Taraxella* sp. [Johor] | d246 | m | Malaysia: Johor: Gunung Belumut | KM033172 |  |  |  | KM032944 |  |  | KM032909 |
| *Taraxella* sp. [Pahang] | d248 | m | Malaysia: Pahang: Cameron Highlands | KM033173 |  |  |  | KM032945 | KM033197 |  |  |
| *Taraxella* sp. [Pahang] | LiD-001-003-06 | f | Malaysia: Pahang: Cameron Highlands |  | KM033106 ^S^ |  |  |  |  | KM033217 ^S^ | KM032910 ^S^ |
| *Yaginumanis wanlessi* Zhang & Li, 2005 | Su et al. 2007 | f | China: Sichuan: Luzhou | EF419050/EF419081* | EF419016* |  |  |  | EF419139* | EF419114* | EF418985/EF419166* |
|  |  |  |  |  |  |  |  |  |  |  |  |
| **Lapsiines** |  |  |  |  |  |  |  |  |  |  |  |
| *Galianora bryicola* Maddison, 2006 | d124 | m | Ecuador: Napo | DQ665771* | DQ665741* | KM033025 |  | EU522706* | DQ665717* | DQ665758* | DQ665727* |
| *Galianora sacha* Maddison, 2006 | d116 | j | Ecuador: Napo | DQ665766* | DQ665734* | KM033026 | KM032968 | EU522707* | DQ665716* | DQ665754* |  |
| *Lapsias canandea* Maddison, 2012 | d442 | m | Ecuador: Esmeraldas: Reserva Canande | KM033174 |  |  |  |  |  |  |  |
| *Lapsias guamani* Maddison, 2012 | UBC-SEM AR00191 | f | Ecuador: Napo: Río Guamani | KM033175 |  | KM033027 |  |  |  |  |  |
| *Lapsias lorax* Maddison, 2012 | UBC-SEM AR00194 | m | Ecuador: Pichincha: Bellavista | KM033176 |  | KM033028 |  |  |  |  |  |
| *Soesiladeepakius lyra* Ruiz & Maddison, 2012 | GR130 |  | Brazil: Amazonas: Manaus | JQ312077 |  | KM033029 |  | JQ312074* |  |  | JQ312079* |
| *Thrandina bellavista* Maddison, 2012 | d396 | m | Ecuador: Pichincha: Bellavista Cloud Forest | KM033177 |  | KM033030 |  |  |  |  |  |
| *Thrandina cosanga* Maddison, 2012 | d395 | m | Ecuador: Napo: Vinillos | KM033178 |  |  |  |  |  |  |  |
| *Thrandina parocula* Maddison, 2006 | d123 | m | Ecuador: Morona Santiago | DQ665779* | KM033107 |  |  | EU522720* | DQ665718* | DQ665761* | DQ665726* |
| *Thrandina parocula* Maddison, 2006 | d394 | f | Ecuador: Napo: Río Guamani |  |  | KM033031 | KM032969 |  |  |  |  |
|  |  |  |  |  |  |  |  |  |  |  |  |
| **Eupoa** |  |  |  |  |  |  |  |  |  |  |  |
| *Eupoa nezha* Maddison & Zhang, 2007 | d220/MRB102 | m | China: Guangxi: Daqingshan Park | EF201648* | EF201666* | KM033032 |  |  |  | EF201668* | EF201667* |
|  |  |  |  |  |  |  |  |  |  |  |  |
| **Hisponines** |  |  |  |  |  |  |  |  |  |  |  |
| cf. *Tomocyrba* sp. [Madagascar] | d305 | f | Madagascar: Toamasina Province | KM032881* |  |  |  |  |  |  |  |
| *Hispo macfarlanei* Wanless, 1981 | d404 | m | Madagascar: Toamasina Province | KM032882* |  |  | KM032970 |  |  |  |  |
| *Hispo* sp. [Madagascar] | d309 | f | Madagascar: Fianarantsoa Province | KM032883* |  |  |  |  |  |  |  |
| *Jerzego* cf. *alboguttatus* Simon, 1903 | SWK12-4787 | j | Malaysia: Sarawak: Lambir Hills | KM032884* |  |  |  |  |  |  |  |
| *Jerzego corticicola* Maddison, 2014 | SWK12-2900 | f | Malaysia: Sarawak: Mulu National Park | KM032885* |  |  |  |  |  |  | KM032887* |
| *Massagris contortuplicata* Wesolowska & Haddad, 2013 | d082 | f | South Africa: Kwazulu-Natal Province | DQ665772* | KM033108 | KM033033 |  |  | DQ665705* |  | DQ665722* |
| *Massagris schisma* Maddison & Zhang, 2006 | d081 | m | South Africa: Northern Cape | DQ665762* | KM033109 | KM033034 |  |  |  |  | DQ665728* |
| *Tomobella andasibe* (Maddison & Zhang, 2006) | d127 | m | Madagascar: Toamasina Province | DQ665780* | DQ665752* | KM033035 |  |  | KM033198 |  | DQ665725* |
| *Tomocyrba* sp. [Madagascar] | d306 | m | Madagascar: Fianarantsoa Province | KM032886* |  |  |  |  |  |  |  |
| *Tomomingi* sp. [Gabon] | MRB243 | f | Gabon: Woleu-Ntem: Monts de Cristal | JX145764* | KM033110 | KM033036 | KM032971 | JX145850* |  | JX145684* |  |
|  |  |  |  |  |  |  |  |  |  |  |  |
| **Salticoida** |  |  |  |  |  |  |  |  |  |  |  |
| **Agoriines** |  |  |  |  |  |  |  |  |  |  |  |
| *Agorius constrictus* Simon, 1901 | d172 | f | Malaysia: Selangor |  |  |  |  | KM032953 |  |  |  |
| *Agorius constrictus* Simon, 1901 | d213 | f | Malaysia: Selangor |  | KM033119 | KM033072 |  |  |  |  | KM032921 |
| *Agorius* sp. [Selangor] | d299 | m | Malaysia: Selangor | KM033189 |  | KM033073 |  |  |  |  |  |
| *Synagelides* cf. *lushanensis* Xie & Yin, 1990 | d214 | f | China: Sichuan |  |  | KM033074 |  |  |  |  |  |
| *Synagelides* cf. *palpalis* Zabka, 1985 | MRB050 | f | China: Guangxi: Pingxiang City |  |  |  |  |  |  |  | KM032922 |
| *Synagelides* cf. *palpalis* Zabka, 1985 | d225 | f | China: Guangxi: Pingxiang City | KM033190 |  |  |  |  |  | KM033226 |  |
|  |  |  |  |  |  |  |  |  |  |  |  |
| **Amycoids** |  |  |  |  |  |  |  |  |  |  |  |
| *Cotinusa* sp. [Ecuador] | MRB024 | m | Ecuador: Morona-Santiago | JX145746* | KM033120 | KM033075 | KM032987 | JX145832* |  | JX145671* | JX145896* |
| *Hurius vulpinus* Simon, 1901 | S213 | f | Ecuador: Quito | AY297239* |  |  |  |  |  | AY297368* | AY296662/AY297306* |
| *Hurius* cf. *vulpinus* Simon, 1901 | d156 | m | Ecuador: East of Gualaceo |  |  | KM033076 |  | EU522712* | KM033203 |  |  |
| *Hypaeus* aff. *miles* Simon, 1900 [Ecuador] | d130 | m | Ecuador: Napo | EU815499* | KM033121 | KM033077 | KM032988 | EU522702* |  |  | KM032923 |
| *Sarinda cutleri* (Richman, 1965) | MRB193 | f | U.S.A.: Arizona, Prescott | JX145744* |  | KM033078 |  | KM032954 |  | JX145669* | JX145895* |
| *Sitticus floricola palustris* (Peckham & Peckham, 1883) | d030 | m | Canada: Nova Scotia | DQ665778* | KM033122 | KM033079 | KM032989 |  | KM033204 | DQ665760* | DQ665729* |
|  |  |  |  |  |  |  |  |  |  |  |  |
| **Astioids** |  |  |  |  |  |  |  |  |  |  |  |
| *Arasia mollicoma* (L. Koch, 1880) | d046 | m | Australia: New South Wales | EU815483* | EU815532* |  | KM032990 | JX145834* | KM033205 | EU815598* | EU815550* |
| *Helpis minitabunda* (L. Koch, 1880) | d265 | m | Papua New Guinea: Enga Province |  | KM033123 | KM033080 | KM032991 | KM032955 |  | KM033227 |  |
| *Ligurra latidens* (Doleschall, 1859) | d175 | m | Singapore | JX145749* |  | KM033081 |  | JX145835* |  |  | JX145898* |
| *Ligurra latidens* (Doleschall, 1859) | LiD-001-027-05 | f | Singapore |  | EF418993* |  |  |  | EF419120* | EF419091* |  |
| *Mopsus mormon* Karsch, 1878 | d018 | m | Australia: Queensland: Cow Bay | EU815470* | EU815529* | KM033082 |  | JX145836* | KM033206 | EU815586* |  |
| *Myrmarachne* sp. [Pahang] | d162 | m | Malaysia: Pahang | EU815507* | KM033124 | KM033083 | KM032992 | JX145837* |  | EU815616* | EU815565* |
| *Neon reticulatus* (Blackwall, 1853) | d283 | m | U.S.A.: Arizona: Mt. Bigelow | KM033191 | KM033125 | KM033084 | KM032993 | KM032956 |  |  |  |
| *Nungia epigynalis* Zabka, 1985 | d221 | f | China: Guangxi, Pingxiang City | KM033192 |  |  |  |  |  |  | KM032924 |
| *Simaetha* sp. | d027 | m | Australia: Queensland | EU815477* | KM033126 | KM033085 |  | JX145839* |  | EU815592* | EU815546* |
| *Trite pennata* Simon, 1885 | d035 | m | New Caledonia: Mt. Koghis | EU815478* |  | KM033086 |  | KM032957 | KM033207 | EU815593* | EU815547* |
|  |  |  |  |  |  |  |  |  |  |  |  |
| **Baviines** |  |  |  |  |  |  |  |  |  |  |  |
| *Bavia* aff. *aericeps* Simon, 1877 [Sabah] | d079 | m | Malaysia: Sabah | EU815490* | KM033127 |  |  | KM032958 |  | EU815603* | KM032925 |
| *Stagetilus* sp. [Selangor] | MRB079 | f | Malaysia: Selangor | KM033193 |  | KM033087 |  | KM032959 |  |  | KM032926 |
|  |  |  |  |  |  |  |  |  |  |  |  |
| **Marpissoids** |  |  |  |  |  |  |  |  |  |  |  |
| *Afromarengo* sp. [Gabon] | MRB262 | m | Gabon: Ngounié: Waka National Park | JX145758* | KM033128 | KM033088 | KM032994 | JX145842* |  | JX145682* | JX145905* |
| *Dendryphantes hastatus* (Clerck, 1757) | d043 | f | Poland: Siedlce | EF201646* | KM033129 | KM033089 |  |  |  | KM033228 | KM032927 |
| *Platycryptus californicus* (Peckham & Peckham, 1888) | d316 | m | Canada: Vancouver | KM033194 |  | KM033090 | KM032995 | KM032960 |  | KM033229 |  |
| *Rhene* sp. [Pahang] | LiD-001-021-05 | f | Malaysia: Pahang: Cameron Highlands | EF419044* | EF419010* |  |  |  | EF419134* | EF419108* | EF418979/EF419160* |
| *Tisaniba mulu* Zhang & Maddison 2014 | SWK12-1244 | f | Malaysia: Sarawak: Mulu National Park | KM032876* |  |  |  |  |  |  | KM032880* |
|  |  |  |  |  |  |  |  |  |  |  |  |
| **Saltafresians** |  |  |  |  |  |  |  |  |  |  |  |
| *Aelurillus* cf. *ater* (Kroneberg, 1875) | d140 | f | Kazakhstan: Almaty Region | EU815504* | EU815536* | KM033037 | KM032972 | JX145831* | KM033199 | EU815615* | EU815564* |
| *Amphidraus complexus* Zhang & Maddison, 2012 | JXZ035 | m | Ecuador: Napo: Jatun Sacha | KC615380* |  | KM033038 |  | KC616069* |  | KC615640* | KC615806* |
| *Athamas* cf. *whitmeei* O. P.-Cambridge, 1877 | JXZ345 | f | Papua New Guinea: New Britain |  |  |  |  | KC616286* |  | KC615649* | KC615822* |
| *Bacelarella pavida* Szüts & Jocqué, 2001 | d195 | m | Ghana: Central Region | EU815511* | EU815538* | KM033039 | KM032973 | KM032946 |  | EU815618* | EU815569* |
| *Bathippus macrognathus* (Thorell, 1881) | JXZ372 | m | Papua New Guinea: Southern Highlands Prov. | KC615407* |  | KM033040 |  | KC616305* |  |  | KC615835* |
| *Bianor maculatus* (Keyserling, 1883) | d017 | f | Australia: South Australia: Glenelg | EU815469* |  | KM033041 |  |  | KM033200 | EU815585* | EU815542* |
| *Bristowia afra* Szüts, 2004 | JXZ363 | m | Gabon: Woleu-Ntem: Monts de Cristal | KC615409* |  |  |  | KC616301* |  |  |  |
| *Bristowia afra* Szüts, 2004 | MRB230 | m | Gabon: Woleu-Ntem: Monts de Cristal |  |  | KM033042 |  |  |  | KM033218 |  |
| *Cheliceroides longipalpis* Zabka, 1985 | d222 | m | China: Guangxi: Tianlin County, Langping |  | KM033111 | KM033043 |  | JX145830* |  | KM033219 | EU815579* |
| *Cheliceroides* cf. *longipalpis* Zabka, 1985 | d415 | m | China: Guizhou: Leigongshan | KM033179 |  |  |  |  |  |  |  |
| *Chinattus parvulus* (Banks, 1895) | d009 | f | U.S.A.: North Carolina | EU815464* | EU815525* | KM033044 |  | JX145848* | KM033201 | EU815581* |  |
| *Chinophrys pengi* Zhang & Maddison, 2012 | JXZ145 | j | China: Guangxi: Tianlin County | KC615416* |  | KM033045 |  | KC616146* |  |  | KC615843* |
| *Corythalia locuples* (Simon, 1888) | JXZ315 | m | Dominican Republic: La Vega | KC615390* |  | KM033046 |  | KC616260* |  | KC615645* | KC615816* |
| *Cosmophasis umbratica* Simon, 1903 | Su et al. 2007 | f | Singapore | EF419020* |  |  |  |  | EF419117* | EF419085* | EF418960/EF419141* |
| *Cytaea nimbata* (Thorell, 1881) | JXZ229 | m | Papua New Guinea: Eastern Highlands Prov. | KC615474* |  | KM033047 |  | KC616197* |  | KC615693* | KC615899* |
| *Diolenius varicus* Gardzińska & Zabka, 2006 | JXZ349 | m | Papua New Guinea: New Britain | KC615480* |  | KM033048 |  | KC616290* |  | KC615695* | KC615905* |
| *Diplocanthopoda marina* Abraham, 1925 | d209 | f | Singapore | KM033180 |  |  |  | KM032947 |  | KM033220 | KM032911 |
| *Eburneana* sp. [Gabon] | MRB231 | j | Gabon: Woleu-Ntem: Monts de Cristal | KM033181 |  | KM033049 |  | JX145858* |  | KM033221 | KM032912 |
| *Echeclus* sp. [Selangor] | MRB089 | m | Malaysia: Selangor | KM033182 |  |  |  | KM032948 |  | KM033222 | KM032913 |
| *Euophrys frontalis* (Walckenaer, 1802) | JXZ137 | m | Germany: Authausen | KC615536* |  | KM033050 |  | KC616139* |  |  | KC615960* |
| *Evarcha proszynskii* Marusik & Logunov, 1998 | d096 | m | Canada: British Columbia: Richmond | DQ665765* | KM033112 |  |  | EU522704* |  |  | DQ665723* |
| *Evarcha proszynskii* Marusik & Logunov, 1998 | d323 | m | Canada: Vancouver |  |  | KM033051 | KM032974 |  |  |  |  |
| *Freya decorata* (C. L. Koch, 1846) | d211 | m | Ecuador: Napo | EU815521* | EU815539* |  | KM032975 | EU522705* |  |  | JX145908* |
| *Gedea* cf. *tibialis* Zabka, 1985 | MRB090 | m | Malaysia: Selangor | KM033183 |  |  |  | KM032949 |  | KM033223 | KM032914 |
| *Habrocestum* cf. *albimanum* Simon, 1901 | d132 | m | South Africa: Western Cape Province | EU815500* |  |  |  |  |  | EU815611* | EU815562* |
| *Habronattus borealis* (Banks, 1895) | d207 | m | Canada: Nova Scotia | KM033184 |  | KM033052 | KM032976 | KM032950 |  | KM033224 | KM032915 |
| *Hasarius adansoni* (Audouin, 1826) | d295 | m | Singapore |  | KM033113 | KM033053 | KM032977 |  |  |  |  |
| *Hasarius adansoni* (Audouin, 1826) | S130/S131/S324 | f | U.S.A.: Hawaii; Isreal | AY297281* |  |  |  |  |  | AY297409* |  |
| *Heliophanus cupreus* (Walckenaer, 1802) | d044 | m | Poland: Mielik | DQ665769* | KM033114 |  |  | EU522710* | DQ665710* | DQ665756* | KM032916 |
| *Idastrandia* cf. *orientalis* (Szombathy, 1915) | d108 | m | Malaysia: Sabah: Mt. Kinabalu | EU815535; EU815496* | EU815535* |  |  | JX145852* |  | EU815608* | EU815560* |
| *Langerra* aff. *longicymbium* Song & Chai, 1991 | d182 | m | Malaysia | KM033185 |  | KM033054 |  |  |  |  | KM032917 |
| *Leptorchestes berolinensis* (C. L. Koch, 1846) | d086 | m | Poland: Lublin | EU815491* | EU815534* | KM033055 |  |  |  | EU815604* | EU815556* |
| *Longarenus brachycephalus* Simon, 1903 | MRB258 | m | Gabon: Estuaire: Mondah Forest | JX145798* |  | KM033056 | KM032978 | KM032951 |  | JX145707* | KM032918 |
| *Nannenus* sp. [Pahang] | d105 | m | Malaysia: Pahang | EU815493* |  | KM033057 | KM032979 | JX145853* |  |  | EU815558* |
| *Naphrys pulex* (Hentz, 1846) | JXZ081 | m | Canada: Ontario: St. Williams | JX145760* | KM033115 |  | KM032980 | JX145844* |  | KC615749* | JX145907* |
| *Omoedus orbiculatus* (Keyserling, 1881) | d008 | m | Australia: Queensland |  |  |  |  |  |  | KC615792* |  |
| *Omoedus orbiculatus* (Keyserling, 1881) | JXZ136 | f | Australia: Queensland, Stradbroke Island | JX145762* | KM033116 | KM033058 |  | JX145846* | KM033202 |  |  |
| *Omoedus papuanus* Zhang & Maddison, 2012 | JXZ286 | m | Papua New Guinea: Eastern Highlands Prov. | KC615619* |  | KM033059 |  | KC616234* |  | KC615790* | KC616042* |
| *Pellenes peninsularis* Emerton, 1925 | d057 | m | Canada: Nova Scotia | DQ665774* | KM033117 | KM033060 |  | JX145864* | DQ665712* |  |  |
| *Pellenes peninsularis* Emerton, 1925 | d400 | f | Canada: Nova Scotia: Barneys River |  |  |  | KM032981 |  |  |  |  |
| *Phaulostylus grammicus* Simon, 1902 | d304 | m | Madagascar: Toamasina Province | KM033186 |  | KM033061 |  |  |  |  |  |
| *Philaeus chrysops* (Poda, 1761) | d025 | f | Italy: Calabria, Gozza | EU815475* | EU815530* | KM033062 |  | JX145855* |  | EU815590* | EU815545* |
| *Phintella* sp. [Gabon] | d402 | m | Gabon: Ngounié | KM033187 |  | KM033063 | KM032982 |  |  |  |  |
| *Plexippus paykulli* (Audouin, 1826) | LiD-001-029-05 | f | Singapore |  | EF419002* |  |  |  | EF419127* |  |  |
| *Plexippus paykulli* (Audouin, 1826) | MRB016 | m | Singapore: Nee Soon Swamp Forest | JX145784* |  | KM033064 |  | EU522713* |  |  |  |
| *Plexippus paykulli* (Audouin, 1826) | S73 |  | U.S.A.: Florida |  |  |  |  |  |  | AY297384* | AY296674/AY297317* |
| *Pochyta* cf. *pannosa* Simon, 1903 | MRB257 | m | Gabon: Ngounié: Waka National Park | JX145806* |  | KM033065 | KM032983 | KM032952 |  | JX145715* | KM032919 |
| *Saitis barbipes* (Simon, 1868) | JXZ147 | f | Spain: Sitges | KC615589* |  | KM033066 |  | KC616147* |  | KC615767* | KC616011* |
| *Salticus scenicus* (Clerck, 1757) | d003 | j | Canada: British Columbia: Mission | DQ665777* | KM033118 | KM033067 | KM032984 | EU522719* | DQ665713* | JX145663* | AY296707/AY297352* |
| *Thiania bhamoensis* Thorell, 1887 | LiD-001-028-05 | f | Singapore | EF419049/EF419080* | EF419015* |  |  |  | EF419138* | EF419113* | EF418984/EF419165* |
| *Trydarssus* cf. *nobilitatus* (Nicolet, 1849) | MRB270 | m | Argentina: Neuquén: Pino Hachado | KM033188 |  | KM033068 | KM032985 | JX145847* |  | KM033225 | KM032920 |
| *Tusitala lyrata* (Simon, 1903) | MRB226 | m | Gabon: Ngounié, Waka National Park | JX145771* |  | KM033069 |  | JX145856* |  | JX145689* | JX145912* |
| *Yllenus arenarius* Menge, 1868 | d013 | j | Poland: Kozki |  | EU815527* |  |  |  |  | EU815583* | EU815541* |
| *Yllenus arenarius* Menge, 1868 | JXZ173 | j | Poland: Kozki | JX145766* |  | KM033070 | KM032986 | JX145851* |  |  |  |
| *Zabkattus furcatus* Zhang & Maddison, 2012 | JXZ218 | m | Papua New Guinea: Central Prov. | KC615503* |  | KM033071 |  | KC616190* |  |  | KC615928* |
